# Customized synthesis of phosphoprotein bearing phosphoserine or its nonhydrolyzable analog

**DOI:** 10.1016/j.synbio.2022.11.004

**Published:** 2022-11-24

**Authors:** Dong Liu, Yingying Liu, Hua-Zhen Duan, Xinjie Chen, Yanan Wang, Ting Wang, Qing Yu, Yong-Xiang Chen, Yuan Lu

**Affiliations:** a Key Laboratory of Industrial Biocatalysis (Ministry of Education), Department of Chemical Engineering, Tsinghua University, Beijing, 100084, China; b Key Laboratory of Bioorganic Phosphorus Chemistry and Chemical Biology (Ministry of Education), Department of Chemistry, Tsinghua University, Beijing, 100084, China

**Keywords:** Cell-free synthetic biology, Phosphoprotein, Phosphorylation, Orthogonal translation system, Nonhydrolyzable analog

## Abstract

Studies on the mechanism of protein phosphorylation and therapeutic interventions of its related molecular processes are limited by the difficulty in the production of purpose-built phosphoproteins harboring site-specific phosphorylated amino acids or their nonhydrolyzable analogs. Here we address this limitation by customizing the cell-free protein synthesis (CFPS) machinery via chassis strain selection and orthogonal translation system (OTS) reconfiguration screening. The suited chassis strains and reconfigured OTS combinations with high orthogonality were consequently picked out for individualized phosphoprotein synthesis. Specifically, we synthesized the sfGFP protein and MEK1 protein with site-specific phosphoserine (O-pSer) or its nonhydrolyzable analog, 2-amino-4-phosphonobutyric acid (C-pSer). This study successfully realized building cell-free systems for site-specific incorporation of phosphonate mimics into the target protein. Our work lays the foundation for developing a highly expansible CFPS platform and the streamlined production of user-defined phosphoproteins, which can facilitate research on the physiological mechanism and potential interference tools toward protein phosphorylation.

## 1 Introduction

Protein phosphorylation is a critical post-translational modification that plays a pivotal role in the regulation of diverse cellular processes, such as cell growth, metabolism, apoptosis, and signal transduction pathways [1]. Protein phosphorylation commonly occurs on serine, threonine, and tyrosine residues. In particular, phosphorylated serine (pSer) has been identified as the most abundant form [1,2]. Dysregulation of protein phosphorylation is implicated in a wide variety of human diseases, and it is emerging as a compelling therapeutic target for disease treatment [3]. A prerequisite for deciphering the function of phosphoproteins and developing effective therapeutic interventions is to generate homogeneous full-length proteins with defined phosphorylation status at high yields. Several biological and chemical approaches are widely applied in producing phosphoproteins but still have their individual limitations [4,5]. Phosphoproteins directly separated from cells and tissues are generally heterogeneous mixtures [6]. Late-stage enzymatic protein modification using kinases has difficulty in controlling the stoichiometry and site-specificity of phosphorylation [7]. The total and semi-chemical protein synthesis based on the native chemical ligation (NCL) are robust methods for phosphoprotein preparation. However, when using such synthetic methods, the installation sites of phosphorylation are often restricted near the protein termini, and the synthetic efficiency is limited by protein's size and structure [[8], [9], [10]]. Recently, the genetic code expansion (GCE) method, which employs a re-engineered cellular protein translation machine, has begun to show promise in overcoming the drawbacks of other approaches by allowing for the site-specific incorporation of phosphorylated amino acids as a whole, including pSer and phosphorylated Tyr, into target proteins [11,12]. Stop codon reassignment, elongation factor refinement and removal, release factor competition minimization, and molecular evolution of the orthogonal aminoacyl tRNA synthetase/tRNA (aaRS/tRNA) pairs have all been used to continuously improve this tactic [[13], [14], [15], [16], [17]]. In the GCE strategy, orthogonality, which relates to the UNAA incorporation efficiency and efficient multiple UNAA incorporation, is the key research aim.

In particular, the *in vitro* GCE strategy using cell-free protein synthesis (CFPS) systems, which exploits crude cell extracts or purified transcription-translation machinery instead of the intact cells to conduct protein expression, has been successfully applied for accessing natural or unnatural proteins. The open nature of the CFPS platform allows for high-level control over protein expression conditions as well as unique freedom in the redesign of engineered orthogonal translation system components, resulting in several advantages [[18], [19], [20]], including: 1) tolerating polar amino acid substrates like phosphorylated Ser that hardly penetrate cell membrane, 2) being compatible with the expression of some cell-toxic proteins, 3) flexibly controlling the system components and reaction environment, 4) facilitating condition screening or prototyping for efficient incorporation of unnatural amino acids into proteins, and 5) easily integrating with other advanced technologies due to open features [[21], [22], [23]]. CFPS technology has been widely applied in versatile fields, including prototyping, biocatalysis, biosensing, and biomedicine. In 2015, Jewett and his coworkers optimized the engineered translation machinery in the CFPS system and first demonstrated the synthesis of active MEK1 harboring the site-specifically incorporated phosphoserines. In this technique, they employed crude extracts from the recoded chassis strain C321ΔA with release factor 1 (RF-1) -deficient, all amber codons lacking and the phosphoserine-specific phosphatase SerB deletion, and utilized the Sep (O-Phosphoserine) orthogonal translation system (Sep-OTS) developed by previous efforts with the increased Sep-tRNA gene copy number from one to five and the combination of OTS components onto one vector [15,24,25]. Nevertheless, fully exploiting the potential of CFPS system as a customized GCE platform for phosphoprotein production via flexible manipulation of protein translation machinery, such as chassis strain selection and OTS reconfiguration screening, remains demanding.

Additionally, owing to the lability of phosphorylated residue toward phosphatase that can catalyze the removal of phosphate group, it is essential to generate phosphatase-resistant phosphoprotein mimetics for *in vivo* functional application [4,5]. However, aspartate/glutamate and thiophosphate substitution neither enable a fully physicochemical properties simulation of the phosphate group nor gain a complete phosphatase resistance [26]. Serine phosphorylation is a reversible reaction respectively catalyzed by kinases and phosphatases, so that the phosphate group can be removed easily from serine by phosphatases. Instead, using nonhydrolyzable methylene (CH_2_) or difluoromethylene (CF_2_) phosphonate to mimic the phosphorylated residue can overcome these limitations [[27], [28], [29]]. CH_2_ moiety, which replaces phosphoryl ester oxygen in pSer, can be incorporated into proteins to avoid cleavage by phosphatases. Thus, the CH_2_-phosphonate analog was embedded into the specific sites of proteins *in Escherichia coli* or in *mammalian cells* via the GCE strategy [[30], [31], [32]]. However, the exogenously added phosphonate analogs need to overcome the limitation of cell permeability, which can benefit from the CFPS system. Therefore, it was also very necessary to apply the flexible CFPS platform into establishing a reengineered protein translation machinery for site-specific incorporation of C-pSer into target proteins.

**Fig. 1:**
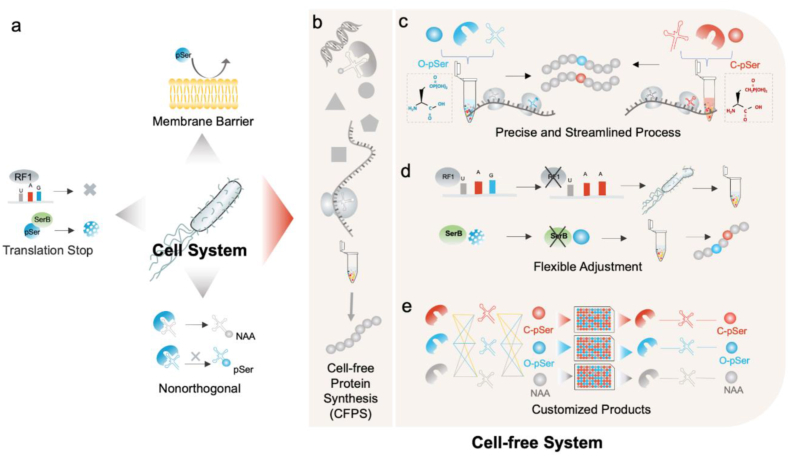
**Flexible CFPS platform with chassis strain selection and OTS reconfiguration screening for the customized synthesis of phosphoprotein.** (a) Limitations of site-specific pSer incorporation by the genetic code expansion (GCE) strategy: poor cell permeability and OTS orthogonality, as well as competition for the target UAG stop codon. (b) Schematic of the process of cell-free protein expression employing crude extracts from the reengineered chassis strains. (c) Precise control of OTS concentration and addition methods on protein expression conditions for the streamlined production of user-defined phosphoproteins. (d) Flexible chassis strains reengineering and chassis strain selection. (e) High-throughput OTS reconfiguration screening in the setting of selected chassis strains.

In this report, we demonstrated a customized cell-free protein synthesis platform by engineering the process of chassis strain selection and OTSs orthogonality screening, enabling phosphoprotein synthesis. First, we developed a flexible platform for effective chassis strains selection, allowing customized individual phosphoprotein production bearing O-pSer or its nonhydrolyzable CH_2_-phosphonate mimics (C-pSer). Second, we explored the orthogonality of OTS components reconfiguration, thereby achieving the optimum embedding of unnatural amino acids (UNAAs). Third, we synthesized the sfGFP protein and MEK1 protein containing site-specific phosphoserine residue or the nonhydrolyzable methylene phosphonates, respectively (Fig. 1). The robustness and versatility of the engineering platform endow the CFPS system with more controllable and greater freedom of redesign. We expect that this customized cell-free protein synthesis platform can facilitate the production of more phosphoproteins and their stable mimetics, which will benefit the exploration of phosphorylation signaling network and the development of interfering molecular tools.

## 2 Materials and methods

### 2.1 Strains and plasmids

A total of 15 *E. coli* chassis cells were selected in this study, including strains of the BL21 series, the Rosetta series, the *E. coli* MG1655 derivatives (rEc.13 and rEc.13. ΔA), and the C321 series (C321, C321. ΔA, and C321. ΔAΔSerB), which were commercially ordered or constructed, as shown in Supplementary Table 1 pET23a vector was mainly used for the plasmid construction, and all plasmids used in this experiment were sequence-verified (Supplementary Table 2). The sfGFP, o-aaRS and o-tRNA sequences were located between the T7 promoter, RBS, ribozyme sequence and T7 termination on the vector pET-23a. All linear PCR products were amplified with pfu high fidelity DNA polymerase. Proteins containing pSer that were encoded by the TAG codon at either position 2 or 23, respectively, were referred to as 2TAG-sfGFP and 23TAG-sfGFP.

### 2.2 Phosphoserine (O-pSer) and its nonhydrolyzable CH2-phosphonate mimic (C-pSer)

Phosphoserine (O-pSer) was purchased from Sigma-Aldrich (No. P0878, CAS No. 407-41-0). Its nonhydrolyzable CH2-phosphonate mimic (C-pSer), 2-amino-4-phosphonobutyric acid, was homemade. The phosphonate pSer mimic C-pSer was synthesized following the previously reported route [27]. The commercially available *N*-Boc protected L-aspartic α-*tert*-butyl ester was first converted to a succinimide ester, followed by reduction to afford protected l-homoserine. After bromo substitution, the bromide was further coupled with diethyl phosphite via Michaelis-Becker reaction, followed by complete deprotection with 9 N HCl to give the desired phosphonate pSer mimic.

### 2.3 Cell extract preparation

First, single colonies were selected and inoculated in 20 mL LB medium and incubated overnight at 30 °C (200 rpm). Then 12.5 mL overnight culture was inoculated in 250 mL 2 × YTP medium (10 g/L Yeast extract, 16 g/L Tryptone, 5 g/L NaCl, 40 mM K_2_HPO4, 22 mM KH_2_PO4) shaking at 30 °C (200 rpm). When the OD_600_ value was 0.6–0.8, the culture was diluted (1:20) and added into a bottle containing 1 L 2 × YTP medium shaking at 30 °C (200 rpm). Monitored the growth status during the cultivation. In the middle and late stages of the logarithmic growth phase (about 5–6 h), centrifuged at 10,000×*g* for 10 min to collect cells. The bacteria were washed with S30A buffer (14 mM Mg-glutamate, 60 mM K-glutamate, 50 mM Tris, pH 7.7) twice, and the bacteria were considered wet weight. 1 mL S30A was added to 1 g of bacteria, and the bacteria were suspended. The bacteria were broken twice with a high-pressure breaker (15,000 Pa). Then broken samples were centrifuged at 4 °C for 30 min (13,000×*g*), 3 μl of 1 M DTT were added to each 1 mL supernatant, and samples were incubated in the dark at 37 °C for 80 min (120 rpm). The dialysis in S30B dialysis buffer (14 mM Mg-glutamate, 60 mM K-glutamate, 5 mM Tris, pH 8.2) was performed at 4 °C for three times, which could remove small molecules in the cell extracts, including residual natural amino acid pSer in the cell extract that might interfere with the UNAA incorporation and verification. At last, the samples were centrifuged, frozen and stored in the refrigerator at −80 °C.

### 2.4 Cell-free protein synthesis reactions

The CFPS reaction was performed in a 1.5 mL EP tube at 30 °C with a final volume of 20 μl. The reaction system included: 2 μl of 10 × salt, 20 mM magnesium glutamate, 0.1 mM PEP, 0.2 μl of T7 RNA polymerase, 0.2 μl of GSH, 0.8 μl of GSSH, 0.8 μl of 19AA, 0.8 μl of NTP, 5 μl of cell extract, DNA template, aaRS, t-DNA, 5 mM UNAA, 2% polyethylene glycol (PEG) 8000, and ddH_2_O. The sfGFP fluorescence was detected by a microplate reader.

### 2.5 Preparation of linear OTS components

Pfu high-fidelity DNA polymerase was used to perform PCR on o-aaRS and o-tRNA [33]. The reaction components included: 38 μl ddH_2_O, 5 μl 10 × pfu buffer, 1 μl dNTPs (10 mM), 1.75 μl template, 2 μl forward primer, 2 μl reverse primer and 0.25 μl pfu polymerase. The program was run at 94 °C for 3 min, followed by 35 cycles of 94 °C for 30 s, 57 °C for 30 s and 72 °C for 2 min/kb. Final extension was running at 72 °C for 10 min and 4 °C forever. After PCR, the bands were confirmed by DNA agarose gel electrophoresis. The entire PCR product was then recovered, and DNA was recovered using ethanol precipitation. The specific method was to add 1/10 volume of sodium acetate and 1/3 of absolute ethanol to the product and place it at −20 °C overnight. Collected the pellet after centrifugation, washed the pellet twice with 70% ethanol, dried the pellet, and added an appropriate amount of water to dissolve the pellet and test the concentration.

### 2.6 Preparation of aaRS proteins

The host cell for aaRS expression was BL21 (DE3) [34]. First, single colonies were selected and inoculated in 10 mL LB liquid medium, at 37 °C, 220 rpm, overnight cultured. The culture was expanded at 5% of the inoculum. At an OD600 of 0.6–0.8, 1000 μl 1 M isopropyl β-*d*-1-thiogalactopyranoside (IPTG) was added to a final concentration of 1 mM. After 9–12 h incubation at 30 °C and 220 rpm, the cultures were pelleted and washed twice with 20 mL lysis buffer (20 mM Na_2_HPO_4__·_12H_2_O, 50 mM NaCl, 30 mM Imidazole, H_3_PO_4_, pH7.4). The lysis buffer was added to resuspend the bacteria, so that the final OD600 value of the bacteria after dilution was 40–60. A high-pressure disruptor was used to repeatedly disrupt the bacteria twice. The lysate was clarified by centrifugation at 4 °C at 12000 rpm for at least 30 min. Following filtration with 0.45 μm water filters, cell lysate was purified by 5 mL EzFast Ni HP column using ÄKTA Prime system, and then was dialyzed against sterile 1 × PBS buffer (pH 7.4) overnight as previously. Then the protein concentrations were determined by using Quick Start Bradford Protein Assay Kit. When necessary, the proteins were concentrated using Amicon Ultra centrifugal device (10 kDa). Finally, 20% (v/v%) sucrose was added to the protein solution, and stored at −80 °C.

### 2.7 Mass spectrometric detection and analysis

One mL of unnatural sfGFP with a C-terminal His-tag was expressed in a 16-well plate, purified using His-tag affinity chromatography, and then concentrated by a Amicon Ultra centrifugal device (10 kDa). Following 10% SDS-PAGE analysis with staining with Coomassie blue dye, the target protein band was cut from the gel and sent to the protein analysis platform at Tsinghua University for mass spectrometry detection (AB Sciex 4800 plus TOF/TOF). Protein Prospector was used to analyze the mass spectrum results (https://prospector2.ucsf.edu/prospector/mshome.htm). The software could analyze the reliability of the protein, and provide information such as the coverage rate of amino acid sequence, the number of peptide segments, the abundance of protein, physicochemical properties, and so on.

### 2.8 Western blot

The expression levels for each protein were monitored by western blot. For western blot, the gel was obtained through the sodium dodecyl sulfate polyacrylamide gel electrophoresis (SDS-PAGE) process. The proteins in the gel were transferred to a low-fluorescence PVDF blotting membrane in a wet blotting procedure (2 h, 100 V, 400 mA). The blotting was then blocked with 5% milk in TBST for 1 h. The blotting was then incubated with the primary anti-His-tag antibody (1:2000 in blocking solution) and washed with 1 × TBST washing solution (3 × 5 min). Then, the membrane was incubated with secondary RPE-goat-anti-mouse-IgG (1:5000 in washing buffer, 60 min) and washed with washing solution (3 × 5 min). Fluorescence detection was performed using the Chemi-Doc MP instrument (Bio-Rad Laboratories). Blots were imaged using an Azure Biosystems c280 imager.

### 2.9 MEK1 kinase activity assay

MEK1 kinase activity was estimated by detecting ERK2 phosphorylation [16]. MEK1 variants were incubated in buffer (50 mM Tris-HCl, 150 mM NaCl, 1 mM DTT, 20% glycerol, 10 mM MgCl_2_ and 1 mM ATP), and then ERK2 substrate was added to the reaction. The reactions were run on SDS–PAGE gels for western blot analysis. The PVDF membrane was blotted with anti-Phos-Erk antibody or anti-His antibody. Signal was detected by enhanced chemiluminescence.

## 3 Results and discussion

### 3.1 Chassis strain selection for developing cell-free phosphoprotein synthesis platform

The yield of cell-free protein synthesis and the incorporation efficiency of UNAAs mostly rely on the fitness of protein translation machinery, particularly on the adaptability of chassis strain and the potency of orthogonal OTS components. Some impressive advances in CFPS system engineering were largely attributed to extensive efforts on chassis organism development [35,36]. To build a reliable and robust cell-free phosphoprotein synthesis platform, chassis strain selection was first conducted with either endogenous or exogenous OTSs to achieve high adaptability to UNAAs insertion, and meanwhile, cell-free reactions were detected to gain the optimum conditions. Initially, TAG codons were introduced into the super-folder GFP (sfGFP) report protein to allow UNAA embedding. L-phosphoserines (O-pSer) and nonhydrolyzable methylene (CH_2_) phosphonate mimics (C-pSer) were used for the site-specific phosphorylation of customized proteins. Because natural amino acid pSer was found in cells, it might interfere with the pSer incorporation and verification. To avoid this problem and develop a reliable approach, dialysis was performed to remove residual natural amino acids in the cell extracts, which could minimize the interference of natural pSer from cells. Meanwhile, the Sep-OTS was chosen as the translation machinery OTS, which has been proved to be effective for the recognition and incorporation of O-pSer into expanded genetically encoded protein [11,13,14,16,30,31]. The incorporation sites were chosen because they could clearly discriminate between the full-length proteins and the truncated proteins: site 2 and site 23, both of which are located in the N-terminal region and outside of the sfGFP 3D structure. 2TAG-sfGFP and 23TAG-sfGFP are sfGFP proteins that have pSer included at codon position 2 or 23, respectively. For a quantitative assessment of the impact of different chassis strains on protein synthesis, the fluorescence intensity of the sfGFP protein that was produced was employed.

**Fig. 2:**
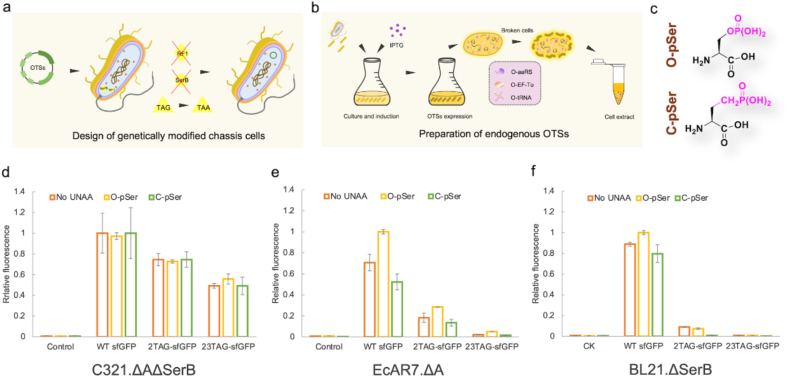
**Chassis strain selection with endogenously expressed OTS.** (a) Design of genetically modified chassis strains with endogenous OTSs system. One-plasmid system included Sep-RS, EF-Sep and Sep-tRNA in the EcAR7. ΔA, C321. ΔAΔSerB and BL21ΔSerB strains. (b) Preparation of endogenous OTSs. The cell extracts containing endogenous OTS system were from genetically recoded strains with one-plasmid OTS system overexpression. (c) The structures of O-pSer and C-pSer. (d–f) The results of Phospho-sfGFP expression during chassis strains selection with endogenous OTSs system in C321. ΔAΔSerB (d), EcAR7. ΔA (e) and BL21. ΔSerB (f) strains. TAG codon at position 2 or 23 directed pSer incorporation into sfGFP, which were named 2TAG-sfGFP or 23TAG-sfGFP. The sfGFP expression was catalyzed by extracts derived from genetically recoded strains, C321. ΔAΔSerB (d), EcAR7. ΔA (e) and BL21. ΔSerB (f). The expression level was determined by fluorescence intensity of generated sfGFP protein. When the relative fluorescence was 1, the corresponding protein expression levels were 1.25 mg/mL (d), 0.58 mg/mL (e), and 2.55 mg/mL (f), respectively. Error bars reported SD from three biological replicates.

To select the top-performing chassis strain adapted to cell-free phosphorylated sfGFP synthesis platform, crude extracts containing the endogenous overexpressed OTS derived from three commonly used chassis strains (*E. coli* BL21ΔSerB, *E. coli* EcAR7ΔA and *E. coli* C321ΔAΔSerB) were exploited (EcAR7: contained seven TAG-to-TAA replacements; C321: all 321 TAGs on their genome were replaced by TAA; ΔA: RF-1 gene was lacking; ΔSerB: SerB gene was knocked out) (Fig. 2a and b, Supplementary Table 1). Besides, to obtain the optimal reaction conditions for cell-free phosphoprotein synthesis system, we conducted the cell-free sfGFP protein synthesis with different magnesium ion concentration gradients and reaction time gradients. A magnesium ion concentration of 20 mM and a 13-h reaction time were found the optimum (Supplementary Figs. S1–4). Among the three chassis strains, although C321. ΔAΔSerB showed better protein-producing performance than EcAR7ΔA and BL21ΔSerB, the protein yield and the O-/C-pSer incorporation ability were not desirable (Fig. 2c–f). 2TAG and 23TAG variants of sfGFP were expressed almost at the same titers in the absence and presence of the UNAA. In the experimental design, cell extracts were made from chassis strains with endogenously expressed OTS components. There might be two reasons causing these results, as shown in Fig. 2. One reason was presumably due to the unknown concentration and difficult regulation of endogenously expressed OTS. The other reason was the low orthogonality of OTS components. Further strategies need to be adopted to improve cell-free unnatural protein synthesis.

**Fig. 3:**
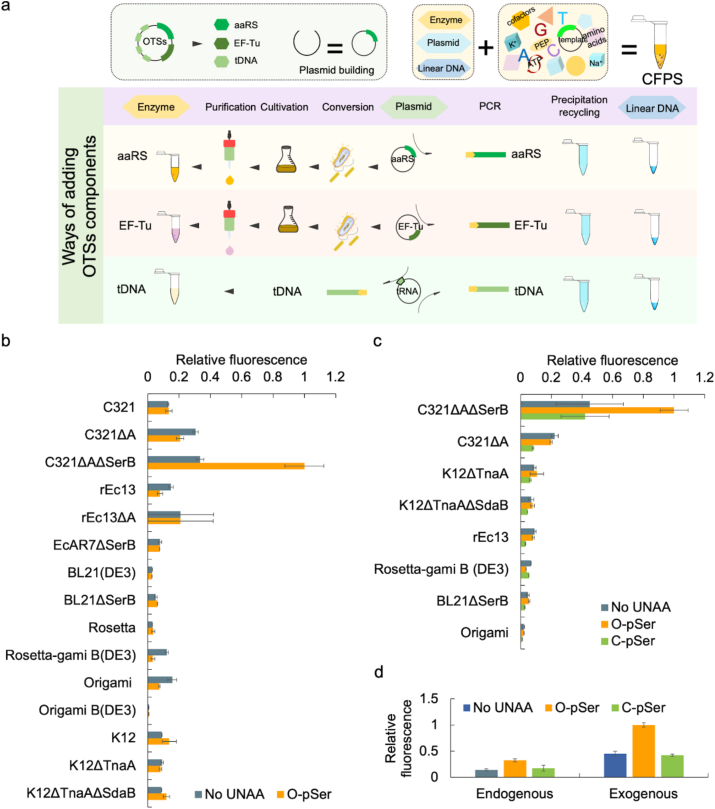
**Chassis strain selection with exogenously added or expressed OTS.** (a) Preparation and purification of OTSs components. (b) The results of O-Phospho-sfGFP expression during chassis strains selection with exogenous OTSs. The cell-free reactions were supplemented with the necessary cell extracts derived from 16 kinds of genetically recoded chassis strains (Supplementary Table 1). TAG codon at position 23 directed O-pSer incorporation into sfGFP, and as a control with no UNAA added. The expression level was determined by fluorescence intensity of generated sfGFP protein. Error bars report SD from three biological replicates. Extended data with a TAG codon at position 2 was shown in Supplementary Fig. S5. (c) The results of O-Phospho-sfGFP and C-Phospho-sfGFP expression, using cell extracts derived from 8 kinds of genetically recoded chassis strains with top performance on O-pSer incorporation. TAG codon at position 23 directed O-pSer and C-pSer incorporation into sfGFP, and as a control with no UNAA added. The expression level was determined by fluorescence intensity of generated sfGFP protein. Error bars report SD from three biological replicates. Extended data with a TAG codon at position 2 was shown in Supplementary Fig. S6. (d) The comparison of sfGFP expression for chassis strains selection with endogenous and exogenous OTSs, respectively. Error bars report SD from three biological replicates.

To improve the UNAAs embedding efficiency along with precise control of OTS concentration, the Sep-OTS components (Sep-RS and Sep-tRNA) were respectively expressed, purified, and further exogenously added into the CFPS system at a definite concentration in the following chassis strain selection and operation process (Fig. 3a). To comprehensively explore the effects of various chassis strains on the cell-free protein synthesis, the selection range of chassis cells was expanded to 15 kinds of commonly used chassis cells (Supplementary Table 1), including strains of the BL21 series (BL21 (DE3) and BL21. ΔSerB) showing high efficiency in expressing genes containing phage T7 promoter (such as pET series), the Rosetta series (Rosetta and Rosetta-Origami B) suitable for pET series vectors and other T7 promoter series vectors with enhanced protein expression levels, the *E. coli* MG1655 derivatives (rEc.13 and rEc.13. ΔA) harboring TAG-to-TAA genomic recoding at seven essential TAG-terminating genes, and the C321 series (C321, C321. ΔA and C321. ΔAΔSerB) with all 321 TAGs on their genome replaced by TAA to facilitate UNAAs embedding.

The screening results of O-pSer embedded in 2TAG-sfGFP and 23TAG-sfGFP showed that C321, C321ΔAΔSerB and BL21ΔSerB chassis strains had better performance for O-pSer embedding (Supplementary Fig. S5 for 2TAG, Fig. 3b for 23TAG). The protein expression yields with O-pSer addition were significantly higher than those without O-pSer addition. The standard curve presenting the relationship between the fluorescent value and the protein expression level was provided in Fig. S6. Meanwhile, C321ΔAΔSerB was the top-performing chassis strain indicated in the screening results of C-pSer embedding in 23TAG-sfGFP (Supplementary Fig. S7 for 2TAG, Fig. 3c for 23TAG). The top performance of C321ΔAΔSerB might be attributed to the reengineered genome of TAG, as well as the loss-of-function of RF-1 and SerB [13,37]. Protein translation process could be terminated when RF-1 recognized the TAG stop codons while the introduction of TAG-to-TAA can prevent the chain termination at the TAG codon. The synergistic effect of RF-1 knockout and TAG-to-TAA reassignment increased the incorporation of O-pSer and C-pSer and improved the phosphoprotein yields.

Notably, the overall yields of CFPS embedding O-pSer or C-pSer using exogenously added or expressed OTS were conspicuously improved compared with those using endogenously overexpressed OTS (Fig. 3d), which manifested the reliability and serviceability of exogenously purified OTS with explicit concentrations. Despite the noticeable higher protein production than endogenous OTS, the exogenous Sep-OTS performed a relatively low efficiency on cell-free sfGFP synthesis embracing C-pSer, thus further improvement of OTS performance for C-pSer incorporation was still needed. Interestingly, the protein expression yields of 23TAG-sfGFP surpassed that of 2TAG-sfGFP, which was probably on account of the position effect. Prior studies have demonstrated that the incorporation efficiency and the synthesis yield of modified protein were dependent on the insertion position of unnatural amino acids [38]. Accordingly, 23TAG-sfGFP instead of 2TAG-sfGFP was applied in the subsequent sfGFP synthesis.

Therefore, chassis strain selection was necessary for directly extending genetically encoded protein phosphorylation to cell-free phosphoprotein synthesis (e.g., O-pSer insertion). Since the importance of chassis strain bias, preliminary chassis strain selection facilitated the discovery of dedicated chassis strains for cell-free phosphoprotein synthesis with high adaptability to designated UNAA incorporation, with the minimum TAG inhibition and RF recognition competition. However, our experimental results also showed that, when a novel purpose-built UNAA (e.g., C-pSer) was introduced into the synthetic protein, only chassis strain selection was inadequate for cell-free protein synthesis with high efficiency and high protein yield, owing to the poor adjustability and orthogonality of OTS components holistic addition. Thereby, a sound and adaptable platform that enables and ensures the precise and flexible manipulation of OTS components is essential for the improved orthogonality of OTS.

### 3.2 Exploration of exogenous OTS addition methods for precise regulation of various OTSs

The essential protein translation machinery includes the aaRS, tRNA, mRNA with anticodon, the ribosome, and some factors like initiation factors (IF) for chain initiation, elongation factors (EF-Tu) for chain elongation, and release factors (RF) for chain termination (Fig. S8a). Some advances, such as RF-1 deletion and stop codon reengineering, have been achieved through chassis strain selection, while the rest of protein translation machinery, like aaRS, tRNA and EF-Tu, required additional improvements as well. As the exogenously adding method displayed precise and flexible operation of Sep-OTS components, to overcome the constraints on new UNAAs adaptability imposed by the holistic addition, we further conducted the investigation on the OTS addition ways and screened the CFPS conditions for accurate OTS control. The adding pattern of translation machinery compositions in the CFPS system could be circular plasmid, linear DNA, or protein. Thus, several aaRS, tRNA and EF-Tu were constructed, expressed, and exogenously purified (Supplementary Tables S2–4, Supplementary Figs. S9–10).

In this case, the adding ways of aaRS into CFPS reaction tube were firstly examined, including adding circular plasmids, linear DNA fragments, and purified enzymes, respectively (Fig. 3a). The expression of aaRS from plasmids and linear DNA in CFPS system were detected by western blotting, but the expression level of aaRS by both methods were very low (Supplementary Fig. S9). Directly adding the aaRS enzyme into the CFPS system was thus decided. The aaRS catalyzes the recognition and binding of an amino acid to its specific tRNA during the translation process. The excessive addition of aaRS has an inhibitory effect on the activity of CFPS, while a deficient amount of aaRS brings about poor embedding efficiency of UNAAs. The screening for the optimal concentration of aaRS in CFPS was then carried out using the purified aaRSs (Supplementary Fig. S11). It was found that the optimal concentration was around 0.03 mM while a high concentration of aaRS had a certain inhibitory effect on CFPS (Fig. S8b). Meanwhile, the optimal concentration of added tDNA was found 75 ng/μl (Supplementary Fig. S12).

In addition, the function of externally added Sep-EF was detected respectively, employing two types of aaRS and tRNA. The results showed that the added EF-Tu had no obvious effect on the CFPS reaction (Fig. S8c and Supplementary Figs. S13–14). It was reasonable that the negatively charged unnatural Sep-tRNA conjugation may be a poor substrate for EF-Tu due to the binding specificity of EF-Tu for the esterified amino acid and the key role of EF-Tu in quality control during protein synthesis [[39], [40], [41]]. In addition, the molecular dynamics simulations and evolutionary analysis also revealed the poor binding pattern between Sep-tRNA and EF-Tu [11,42]. Hence, the EF-Tu was no longer added in our subsequent experiments to reduce the burden of the CFPS system.

All in all, it can be concluded that the holistic OTS addition method was not amenable to the application for new UNAAs incorporation into proteins based on CFPS platform due to the individual optimal reaction concentration of each OTS component. Simply put, when studying the cell-free synthesis of a new UNAA embedded phosphoprotein with exogenously expressed OTS, it was better to add OTS components separately at each optimal concentration instead of introducing the OTS combination. This was very useful for building a credible and resilient cell-free phosphoprotein synthesis platform with highly UNAAs expandability.

### 3.3 Exogenous OTSs components reconfiguration screening for optimum orthogonality

Since adding the detached OTS composition into CFPS reaction was proved a feasible way for flexible OTS operation, to develop a highly efficient and adaptive way of OTS orthogonality enhancement, the reconfiguration of existing OTS components was proposed based on the flexibility and open nature of CFPS. Our research was then expanded to the engineered high-throughput screening of exogenous OTSs components for the optimum OTSs orthogonality, which could enable the customized site-specific phosphoprotein synthesis. The OTS orthogonality screening was performed with twelve kinds of purified aaRSs and seventeen kinds of tRNAs, which possessed essential structures engaged in protein translation through molecular evolution and were commonly used for various UNAAs incorporation in CFPS reaction (Supplementary Tables S3–4 and Figs. S15–16). Meanwhile, 23TAG-sfGFP was employed as a reporter protein to evaluate the insertion efficiency of O-pSer and C-pSer.

To investigate the effects of reconfigured OTS orthogonality on O-pSer and C-pSer embedding, the cell-free expression of 23TAG-sfGFP with O-pSer or C-pSer addition was respectively measured. The relatively high fluorescence value of RS10-T14 and RS9-T12 OTS combinations in 23TAG-sfGFP protein expression indicated that they were effective in both O-pSer and C-pSer incorporation, even better than the originally used Sep-RS/Sep-tRNA (RS0-T0) pair (Supplementary Figs. S17–18). The consistency of OTS reconfiguration for 23TAG-sfGFP synthesis containing either O-pSer or C-pSer was probably resulted from the structural similarity of O-pSer and C-pSer. Thus, these screening results would serve as a guidance for the precise regulation of OTSs components to achieve optimal CFPS performance. Besides, through the contrastive analysis between the reconfigured OTS components and the holistic ones, the protein expression level of sfGFP had a significant improvement, no matter containing the insertion of O-pSer or C-pSer.

**Fig. 4:**
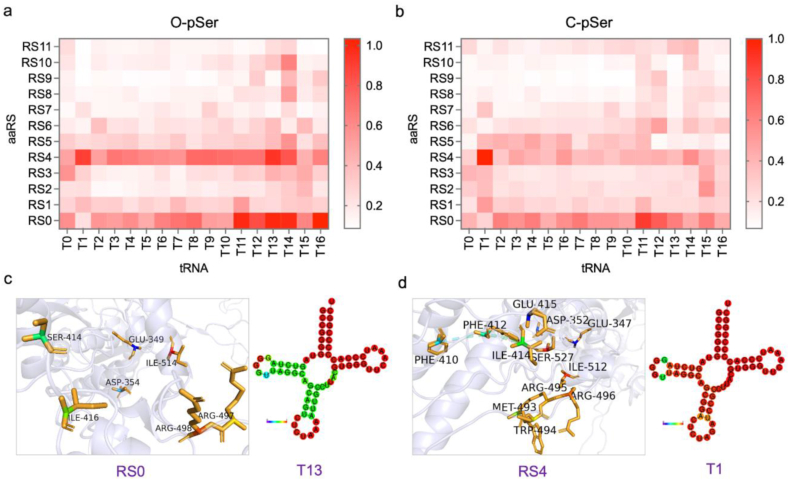
**High-throughput screening of exogenous OTSs reconfiguration orthogonality.** (a) (b) The orthogonality analysis for reconfigured OTS components (aaRS and tRNA for O-pSer or C-pSer incorporation). The heatmap depicted the fluorescence value ratio of the expressed sfGFP (UNAA-added CFPS reaction) to the sfGFP expression baseline (non UNAA-added CFPS reaction). The cell-free reactions were supplemented with the necessary cell extracts derived from genetically recoded C321. ΔAΔSerB strain. A TAG codon at position 23 directed O-pSer (a) and C-pSer (b) incorporation into sfGFP. (c) Structure and sequence analysis of RS0-T13 pair for top-performing O-pSer incorporation. (d) Structure and sequence analysis of RS4-T1 pair for top-performing C-pSer incorporation. The tRNA secondary structure was predicted by RNAfold webserver, and MFE structure drawing encoding base-pair probabilities was shown.

To calibrate the embedding efficiency of O-pSer and C-pSer as well as lucubrate the reconfigured OTS orthogonality rigorously, the measured fluorescence value of expressed sfGFP (UNAA-added CFPS reaction) were compared with the fluorescence value of sfGFP expression baseline (no UNAA-added CFPS reaction, Supplementary Fig. S19). The corresponding fluorescence value ratio of these two datasets were mapped (Fig. 4a and b). It was found that RS0-T13 combination had an outstanding performance for O-pSer embedding, while RS4-T1 combination had relatively high embedding efficiency for C-pSer. The embedding efficiency of C-pSer, though lower than, was consistent with that of O-pSer on general trend, presumably owing to the similar structure of the two UNAAs.

To confirm the insertion of O-pSer and C-pSer into sfGFP, mass spectrometry was performed to detect the resulting sfGFP protein with a high expression yield. The expressed 23TAG-sfGFP embedding O-pSer or C-pSer were purified by gravity column (Supplementary Fig. S20), and the samples were detected through mass spectrometry (Supplementary Figs. S21–22). The mass spectrometry results demonstrated the successful incorporation of O-pSer and C-pSer into sfGFP. However, in the mass spectrometry analysis, only parts of peptide fragments contained O-pSer or C-pSer. The incorporation ratios for O-pSer and C-pSer were 2.72% and 0.80%, respectively (Tables S5–6). It demonstrated that mis-encoding of natural amino acids still happened. Based on protein expression level and mass spectrometry analysis, titers of O-pSer and C-pSer containing sfGFP were estimated to be 18.7 μg/mL and 2.3 μg/mL, respectively. With this cell-free technology platform, more OTS components and new approaches need to be developed or tested to further improve the pSer incorporation efficiency.

To better understand the molecular basis of Sep-RS/tRNA structure for UNAA incorporation, the sequence and structure of standout OTSs components were analyzed. It was found that mutations on tRNAs with top performance mostly occur in the anticodon ring (region 29–42) and the receiving stem of the terminal amino acids. This finding was in accordance with previous studies on classic Sep-RS/tRNA^Cys^_GCA_ structure that Sep-RS recognizes the anticodon (G34, C35 and A36) region of tRNA^Cys^_GCA_ [43]. It was noteworthy that the outstanding OTSs for O-pSer (RS0-T13) and C-pSer (RS4-T1) had a subtle difference in the sequence and structure of tRNA (Fig. 4c and d). As for the aaRSs, mutation sites of the top performed ones were mainly located in the binding sites towards tRNA anticodon. Particularly, the two mutations E417W, F529E, were consistent with previous studies on Sep-RS structure that six residues (Glu412, Glu414, Lys417, Pro495, Ile496 and Phe529), especially Lys417 and Phe529, were critical residues in the vicinity of the tRNA anticodon loop [30]. There was some slight difference on aaRS structure suitable for O-pSer or C-pSer severally, most noticeably a distinction in the tRNA anticodon binding sites between SepRS (RS0) for O-pSer and RS4 for C-pSer (Fig. 4c and d). Previously report showed that the molecular evolution of Sep-tRNA in anticodon stem and loop region, as well as the Sep-RS in anticodon binding region, enabled dramatic improvement of pSer incorporation [14,30]. Our results indicate that these mutation sites were essential for Sep-RS/tRNA genetic modification to improve UNAAs incorporation efficiency. In addition to demonstrating the general mutation sites of OTS consistent with previous studies, our results paid further attention to the subtle OTS structure distinction between O-pSer and C-pSer.

Thus far, a cell-free phosphoprotein synthesis platform capable of user-defined chassis strain selection and OTS orthogonality screening was developed, which avoided complicated genomic molecular evolution of OTS and had the pliability and expandability for diverse UNAAs incorporation. In summary, when a new purpose-built UNAAs was to be incorporated into customized protein, de novo development of CFPS platform demands a robust and resilient modular optimization system for improved OTS orthogonality, flexible UNAAs malleability, and easily user-defined phosphoprotein accessibility. Compared with intricate and time-consuming molecular evolution, the reconfiguration of existing OTS components can promptly obtain competent OTS combinations. In addition, the subsequent sequence and structure analysis of the selected OTS pair revealed the key sites involved in UNAA adaptability to cell-free protein synthesis, which can facilitate further rational directed molecular evolution for new UNAA embedding.

### 3.4 Cell-free synthesis of site-specifically phosphorylated MEK1 kinase

Mitogen-activated protein kinase kinase 1 (MEK1) plays a crucial part of the RAS-RAF-MEK-ERK pathway (or ERK pathway), whose phosphorylation involves various cellular processes, including differentiation, proliferation, motility, apoptosis, and angiogenesis. Targeting MEK1 has become an important therapeutic strategy [[44], [45], [46]]. To further demonstrate the practicability of our customized cell-free phosphoprotein synthesis platform, site-specifically phosphorylated MEK1 kinase was produced by this strategy, and its kinase activity was measured. MEK1 kinase, a key protein in cellular signal transduction, can activate its effector proteins when it is in the active form bearing phosphorylation at serine 217 or serine 221. In this study, we generated the MEK1 protein embedded with O-pSer or C-pSer by selecting the matched chassis strain extracts and using the exogenously added optimal OTSs combination. The resulting MEK1 proteins were detected by western blot.

Chassis strains were individually specific toward different customer proteins, and thus 16 chassis strains mentioned above were investigated to match the synthesis of site-specifically phosphorylated MEK1 (Supplementary Fig. S23). Western blot was conducted to assess the total expression level of MEK1. Finally, it was found that BL21ΔSerB could serve as an ideal chassis strain for MEK1 synthesis.

**Fig. 5:**
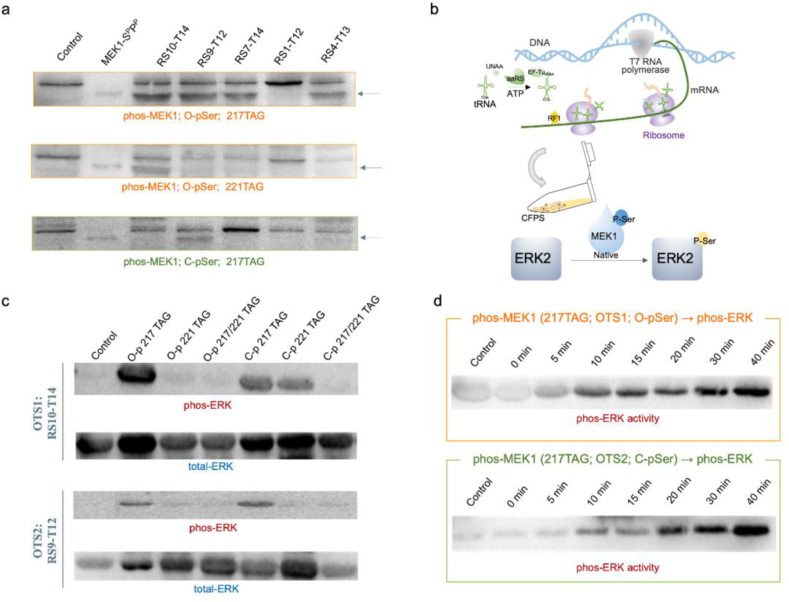
**Cell-free synthesis and activity assessment of site-specifically phosphorylated MEK1.** (a) Quantitation analysis of site-specifically phosphorylated MEK1 production by western blot. (b) Schematic of the process of phosphorylated MEK1 production via cell-free reaction and the subsequent *in vitro* kinase assay using the native MEK1 substrate ERK2. (c) *In vitro* MEK1 kinase activity was assayed using selected OTS reconfiguration and using ERK2 as a substrate. The far-left lane was background ERK2 phosphorylation without MEK1 protein addition. Total ERK was determined by western blot analysis as a control. (d) The time points of *in vitro* MEK1 kinase activity were measured at 0, 5, 10, 15, 20, 30, and 40 min.

With the ideal chassis strain BL21ΔSerB, the OTS components orthogonality screening was implemented for both O-pSer and C-pSer incorporation using several foregoing OTS combinations with comparatively high embedding efficiency (Supplementary Fig. S24). It was found that O-pSer could be embedded into 217-MEK1 protein by using four OTS combinations (RS10-T14、RS9-T12、RS7-T14、RS4-T13) and be embedded into 221-MEK1 protein by using only one OTS combination (RS10-T14). Meanwhile, C-pSer could be embedded into 217-MEK1 with RS9-T12 (Fig. 5a). These results confirmed the personalization capabilities of different OTS reconfiguration for distinct protein synthesis, indicating the significance and necessity of the existing OTS components recombination. By the quantification analysis of the MEK1 protein expression level through gray-scale analysis, the maximum concentration of total MEK1 expression was 1.2 μg/mL. The reconfiguration of existing OTS components realized the rapid response to purpose-built phosphoprotein synthesis, displaying unique features of high operating efficiency and streamlined protein synthesis applicability. It was compatible with the existing OTS components and easy to deploy and utilize, bypassing the maladaptation of UNAAs insertion to achieve high UNAAs embedding flexibility.

To assess the kinase activity of synthesized MEK1, the phosphorylation of ERK2, which is the substrate of MEK1 and can be activated by serine 217 or serine 221 phosphorylated MEK1 protein, was detected by *in vitro* kinase cascade experiments (Fig. 5b). The non-, 217- and 221- phosphorylated MEK1 protein were respectively synthesized by the CFPS strategy via two highest performance OTS combinations--RS10-T14 (named OTS1) and RS9-T12 (named OTS2) bearing O-pSer or C-pSer embedding. The generated phosphorylated MEK1 protein was incubated with a full-length ERK2 protein with greatly reduced autophosphorylation activity. The phosphorylation level of ERK2 catalyzed by MEK1 was detected by western blot using a specific antibody against phosphorylated ERK and an antibody against total ERK (Fig. 5c). The western blot results indicated that ERK2 could be phosphorylated by single-phosphorylated (particularly the 217-phosphorylated) MEK1 kinase synthesized by both OTS combinations (OTS1 and OTS2) bearing O-pSer or C-pSer embedding. This indicated that single-phosphorylation of MEK1 was necessary and sufficient for its proper kinase activity, consistent with previous report [24]. The successful incorporation of the novel phosphoserine analog (C-pSer) in MEK1 was also validated, which was the first demonstration of C-pSer embedding in MEK1 protein via cell-free protein synthesis platform. Meanwhile, the ERK2 phosphorylation catalyzed by MEK1 kinase bearing either O-pSer or C-pSer was completed within about 30 min (Fig. 5d).

Thus, by expanding the genetic code phosphoprotein synthesis in the cell-free platform, sfGFP and MEK1 protein containing site-specific O-pSer or C-pSer were successfully produced utilizing optimum OTS orthogonality and high malleable chassis strains. Compared with commonly adopted molecular evolution and genetic engineering methods for CFPS platform building, a more robust, yet adaptable cell-free framework was constructed by taking a highly modular optimization approach both in design and implementation, embracing chassis strain selection and reconfiguring the finite existing OTS components.

## 4 Conclusion

The main research aim is to build a customized CFPS platform for phosphoprotein, by engineering the process of chassis strain selection and OTSs orthogonality screening. The incorporation of O-pSer and nonhydrolyzable C-pSer are used as examples for this platform. We discovered suitable chassis strains for phosphorylated sfGFP and MEK1 protein synthesis, respectively, as well as several effective OTS recombinations for O-pSer or C-pSer incorporation. Meanwhile, through the sequence and structure analysis of the performed OTS combinations, some new subtle structural features were revealed distinguishing for O-pSer and C-pSer embedding, besides the consistency with previously reported OTS essential structure involved in phosphoprotein synthesis.

Our method demonstrates the capacity to quickly produce phosphoprotein based on the reassembly of existing protein translation components through the exogenously flexible modification and the mutual orthogonality screening of the protein translation machinery in CFPS. Two issues with this technology platform still need to be resolved in their current state, nevertheless. The first is that a powerful function screening system must be integrated with this platform. Mass spectrometry analysis is now utilized to check whether phosphoserine or an analog of it was incorporated into the target protein, however it is not ideal for quick and simple function screening. It is necessary to create additional function verification strategies, such as phos-tag gel electrophoresis. The second is that this platform still needs to be improved before it can be used for industrial manufacturing. Although this cell-free technological platform has shown to be a useful tool for prototyping, it still has limitations for manufacturing, particularly those related to cost and quality assurance. There are two possible approaches. One is using a prototyping screening platform to identify the best OTS components, and then transferring those components to cell systems. The other is improving the method of cell-free preparation to cut costs and maintain reliable quality control.

Looking forward, this cell-free protein synthesis platform, equipped with the adaptability screening of protein synthesis machinery and function verification system, provides the possibility for the technology expansion of CFPS to allow the site-specific incorporation of various purpose-built UNAAs, the customized establishment of optimum protein synthesis devices, and the rapid production of engineered protein.

## Ethics approval

This article does not contain any studies with human participants or experimental animals performed by any of the authors.

## CRediT authorship contribution statement

**Dong Liu:** Investigation, Writing – original draft. **Yingying Liu:** Investigation, Writing – original draft. **Hua-Zhen Duan:** Investigation. **Xinjie Chen:** Investigation. **Yanan Wang:** Investigation. **Ting Wang:** Investigation. **Qing Yu:** Data curation, Writing – original draft. **Yong-Xiang Chen:** Writing – review & editing, Supervision, Project administration, Funding acquisition. **Yuan Lu:** Writing – review & editing, Supervision, Project administration, Funding acquisition.

## Declaration of competing interest

The authors declare that they have no known competing financial interests or personal relationships that could have appeared to influence the work reported in this paper.
