# Supplementary material for: Customized synthesis of phosphoprotein bearing phosphoserine or its nonhydrolyzable analog
Source: Synth Syst Biotechnol. 2022 Nov 24;8(1):69–78. doi: 10.1016/j.synbio.2022.11.004 (PMC9719085; doi:10.1016/j.synbio.2022.11.004)
Supplement: Multimedia component 1 [file mmc1.docx]

Supplementary Information

**Customized synthesis of phosphoprotein bearing phosphoserine or its nonhydrolyzable analog**

Dong Liu^1,#^, Yingying Liu^1,#^, Hua-Zhen Duan^2^, Xinjie Chen^1^, Yanan Wang^1^, Ting Wang^1^, Qing Yu^2^, Yong-Xiang Chen^2,*^ & Yuan Lu^1,*^

1 Key Laboratory of Industrial Biocatalysis (Ministry of Education), Department of Chemical Engineering, Tsinghua University, Beijing 100084, China.

2 Key Laboratory of Bioorganic Phosphorus Chemistry and Chemical Biology (Ministry of Education), Department of Chemistry, Tsinghua University, Beijing 100084, China.

# These authors contributed equally.

* Corresponding authors. Email: chen-yx@tsinghua.edu.cn (Yong-Xiang Chen); yuanlu@tsinghua.edu.cn (Yuan Lu).

**Table of Contents**

[Supplementary Tables S2](#_Toc101726467)

[Supplementary Figures S11](#_Toc101726468)

# Supplementary Tables

**Table S1** Chassis cells selected by UNAA incorporation

| Name | Feature | Source |
| --- | --- | --- |
| *E. coli* C321 | Replace all UAG with UAA, but wild type UAG termination function is not changed. | Addgene #48999 |
| *E. coli* C321.ΔA | Replace all UAG with UAA and removed RF1 (UAG termination function removed). | Addgene #48998 |
| *E. coli* C321.ΔAΔSerB | Replace all UAG with UAA and knockout of phosphoserine transferase gene | Addgene #68306 |
| *E. coli* rEc.13 | The13 occurrences of the amber stop codon have been reassigned to the synonymous TAA codon from MG1655. | Addgene #69494 |
| *E. coli* rEc.13.ΔA | The13 occurrences of the amber stop codon have been reassigned to the synonymous TAA codon from MG1655, and *prfA* deleted. | Addgene #69495 |
| *E. coli* EcAR.7.ΔSerB | Replace seven UAG with UAA and knockout of phosphoserine transferase gene | Addgene #52055 |
| *E. coli* BL21(DE3) | lon and ompT protease deficient | Commercial |
| *E. coli* BL21.ΔSerB | Knockout of phosphoserine transferase gene | Addgene #34929 |
| *E. coli* Rosetta(DE3) | Contains rare codons from eukaryotic cells (AUA,AGG,AGA,CAU,CCC,GGA) | Commercial |
| *E. coli* Rosetta-gami(DE3) | *TrxB* and *Gor* gene mutations and rare codons in eukaryotic cells | Commercial |
| *E. coli* Origami | *trxB* and *gor* from k12 cells contain mutations | Commercial |
| *E. coli* Origami B(DE3) | *trxB/gor* mutant, greatly facilitates cytoplasmic disulfide bond formation | Commercial |
| *E. coli* K12 | Clear genetic information and widely used in DNA recombination experiments | Commercial |
| *E. coli* K12.ΔTnaA | Knockout of tryptophanase gene | This study |
| *E. coli* K12.ΔTnaAΔSdaB | Knockout of tryptophanase and serine deaminase genes | This study |

**Table S2** Strains and plasmid information

| *E. coli* | Resistance | Plasmid Name | Expressed genes |
| --- | --- | --- | --- |
| DH5α | - | - | - |
| DH10B | - | - | - |
| BL21(DE3) | - | - | - |
| BL21 Star(DE3) | - | - | - |
| DH5α | Low-kan | pKD-SepOTSλ | SepRS9/EFSep21, 4xtRNA-Sep-A37 |
| Top 10 | Amp | MEK1-217TAG | N-MEK1-C-his-217TAG |
| Top 10 | Amp | MEK1-221TAG | N-MEK1-C-his-221TAG |
| Top 10 | Amp | MEK1-217\221TAG | N-MEK1-C-his-217\221TAG |
| DH10B | Amp | pET23a-sfGFP(2TAG)-C-His | sfGFP(2TAG) |
| DH10B | Amp | pET23a-sfGFP(23TAG)-C-His | sfGFP(23TAG) |
| DH10B | Amp | pET23a-CH-sfGFP | sfGFP |
| DH10B | Amp | pET23a-SepRS-C-His | SepRS (RS0) |
| DH10B | Amp | pET23a-EF-Tu-C-His | EF-Tu |
| DH10B | Amp | pET23a-pAzFRS-C-His | pAzFRS |
| DH10B | Amp | pET23a-pBpRS-C-His | pBpRS |
| DH10B | Amp | pET23a-pAcFRS-C-His | pAcFRS |
| DH10B | Amp | pET23a-pPaFRS-C-His | pPaFRS |
| DH10B | Amp | pET23a-RS-1-C-His | RS-1 |
| DH10B | Amp | pET23a-RS-2-C-His | RS-2 |
| DH10B | Amp | pET23a-RS-3-C-His | RS-3 |
| DH10B | Amp | pET23a-RS-4-C-His | RS-4 |
| DH10B | Amp | pET23a-RS-5-C-His | RS-5 |
| DH10B | Amp | pET23a-RS-6-C-His | RS-6 |
| DH10B | Amp | pET23a-RS-7-C-His | RS-7 |
| DH10B | Amp | pET23a-RS-8-C-His | RS-8 |
| DH10B | Amp | pET23a-RS-9-C-His | RS-9 |
| DH10B | Amp | pET23a-RS-10-C-His | RS-10 |
| DH10B | Amp | pET23a-RS-11-C-His | RS-11 |
| DH10B | Amp | pET23a.Δrop.Δf1.ori-otDNA | tRNA-0 |
| DH10B | Amp | pET23a.Δrop.Δf1.ori-otDNA | tRNA-1 |
| DH10B | Amp | pET23a.Δrop.Δf1.ori-otDNA | tRNA-2 |
| DH10B | Amp | pET23a.Δrop.Δf1.ori-otDNA | tRNA-3 |
| DH10B | Amp | pET23a.Δrop.Δf1.ori-otDNA | tRNA-4 |
| DH10B | Amp | pET23a.Δrop.Δf1.ori-otDNA | tRNA-5 |
| DH10B | Amp | pET23a.Δrop.Δf1.ori-otDNA | tRNA-6 |
| DH10B | Amp | pET23a.Δrop.Δf1.ori-otDNA | tRNA-7 |
| DH10B | Amp | pET23a.Δrop.Δf1.ori-otDNA | tRNA-8 |
| DH10B | Amp | pET23a.Δrop.Δf1.ori-otDNA | tRNA-9 |
| DH10B | Amp | pET23a.Δrop.Δf1.ori-otDNA | tRNA-10 |
| DH10B | Amp | pET23a.Δrop.Δf1.ori-otDNA | tRNA-11 |
| DH10B | Amp | pET23a.Δrop.Δf1.ori-otDNA | tRNA-12 |
| DH10B | Amp | pET23a.Δrop.Δf1.ori-otDNA | tRNA-13 |
| DH10B | Amp | pET23a.Δrop.Δf1.ori-otDNA | tRNA-14 |
| DH10B | Amp | pET23a.Δrop.Δf1.ori-otDNA | tRNA-15 |
| DH10B | Amp | pET23a.Δrop.Δf1.ori-otDNA | tRNA-16 |

**Table S3** tDNA sequence information

| Name | DNA sequences |
| --- | --- |
| T0 | gccggggtagtctaggggttaggcagcggactctaaatccgccttacgtgggttcaaatcccacccccggct |
| T1 | gccggggtagtctaggggttaggcagcggactctagatccgccttacgtgggttcaaatcccacccccggct |
| T2 | gccggggtagtctaggggctaggcagcggactctagatccgccttacgtgggttcaaatcccacccccggct |
| T3 | gccggggtagtctaggggctaggcagctttctctaaaaaagccttacgtgggttcaaatcccacccccggct |
| T4 | gccggggtagtctaggggctaggcagccttctctaaaagggccttacgtgggttcaaatcccacccccggct |
| T5 | gccggggtagtctaggggctaggcagcaatctctaaaattgccttacgtgggttcaaatcccacccccggct |
| T6 | gccggggtagtctaggggctaggcagcagtctctaaaattgccttacgtgggttcaaatcccacccccggct |
| T7 | gccggggtagtctaggggctaggcagcggcctctaaaaccgccttacgtgggttcaaatcccacccccggct |
| T8 | gccggggtagtctaggggctaggcagcggtctctaaaactgccttacgtgggttcaaatcccacccccggct |
| T9 | gccggggtagtctaggggctaggcagcgacctctaaagctgccttacgtgggttcaaatcccacccccggct |
| T10 | gccggggtagtctaggggttaggcagcggactctagatccgccttacgtgggttcaaatcccacccccggct |
| T11 | gccggggtagtctaggggttaggcagcggactgcagatccgccttacgtgggttcaaatcccacccccggct |
| T12 | gccggcggtagttcagcagggcagaacggcggactctaaatccgcatggcgctggttcaaatccggcccgccgga |
| T13 | gccggggtagtctaggggttaggcagcagtctctaaaattgccttacgtgggttcaaatcccacccccggct |
| T14 | gcccggatggtggaatcggtagacacaagggattctaaatccctcggcgttcgcgctgtgcgggttcaagtcccgctccgggta |
| T15 | gccaggaaacctgatcatgtagatcgaatggactctaaatccgttcagccgggttagattcccggggtttccgcca |
| T16 | ccggcggtagttcagcagggcagaacggcggactctaaatccgcatggcaggggttcaaatcccctccgccggacca |

**Table S4** aaRS sequence information

| Name | DNA sequences |
| --- | --- |
| RS0 | atggcaatgtttaaaagagaagaaatcattgaaatggccaataaggactttgaaaaagcatggatcgaaactaaagaccttataaaagctaaaaagataaacgaaagttacccaagaataaaaccagtttttggaaaaacacaccctgtaaatgacactattgaaaatttaagacaggcatatcttagaatgggttttgaagaatatataaacccagtaattgtcgatgaaagagatatttataaacaattcggcccagaagctatggcagttttggatagatgcttttatttagcgggacttccaagacctgacgttggtttgagcgatgaaaaaatttcacagattgaaaaacttggaattaaagtttctgagcacaaagaaagtttacaaaaaatacttcacggatacaaaaaaggaactcttgatggtgacgatttagttttagaaatttcaaatgcacttgaaatttcaagcgagatgggtttaaaaattttagaagatgttttcccagaatttaaggatttaaccgcagtttcttcaaaattaactttaagaagccacatgacttcaggatggttccttactgtttcagacctcatgaacaaaaaacccttgccatttaaactcttttcaatcgatagatgttttagaagagaacaaaaagaagataaaagccacttaatgacataccactctgcatcctgtgcaattgcaggtgaaggcgtggatattaatgatggaaaagcaattgcagaaggattattatcccaatttggctttacaaactttaaattcattcctgatgaaaagaaaagtaaatactacacccctgaaacacagactgaagtttacgcataccacccaaaattaaaagaatggctcgaagttgctacatttggagtatattcgccagttgcattaagcaaatacggaatagatgtacctgtaatgaatttgggtcttggtgttgaaagacttgcaatgatttctggaaatttcgcagatgttcgagaaatggtatatcctcagttttacgaacacgaacttaatgaccgggatgtcgcttcaatggtaaaactcgataaagttccagtaatggatgaaatttacgatttaacaaaagaattaattgagtcatgtgttaaaaacaaagatttaaaatccccttgtgaattagctattgaaaaaacgttttcatttggaaaaaccaagaaaaatgtaaaaataaacattttttcaaaaatagaaggtaaaaatttactcggaccttcaattttaaacgaaatctacgtttacgatggaaatgtaattggaattcctgaaagctttgacggagtaaaagaagaatttaaagacttcttagaaaaaggaaaatcagaaggggtagcaacaggcattcgatatatcgatgcgctttgctttaaaattacttcaaaattagaagaagcatttgtgtcaaacactactgaattcaaagttaaagttcgacgtgtcagaagtttaagcgacattaacttaaaaatcgatgatatcgcaataaaacagatcatgagcaaaaataaagtaatcgacgttagaggcccagtctttttaaatgtcgaagtaaaaattgaataa |
| RS1 | atggcaatgtttaaaagagaagaaatcattgaaatggccaataaggactttgaaaaagcatggatcgaaactaaagaccttataaaagctaaaaagataaacgaaagttacccaagaataaaaccagtttttggaaaaacacaccctgtaaatgacactattgaaaatttaagacaggcatatcttagaatgggttttgaagaatatataaacccagtaattgtcgatgaaagagatatttataaacaattcggcccagaagctatggcagttttggatagatgcttttatttagcgggacttccaagacctgacgttggtttgagcgatgaaaaaatttcacagattgaaaaacttggaattaaagtttctgagcacaaagaaagtttacaaaaaatacttcacggatacaaaaaaggaactcttgatggtgacgatttagttttagaaatttcaaatgcacttgaaatttcaagcgagatgggtttaaaaattttagaagatgttttcccagaatttaaggatttaaccgcagtttcttcaaaattaactttaagaagccacatgacttcaggatggttccttactgtttcagacctcatgaacaaaaaacccttgccatttaaactcttttcaatcgatagatgttttagaagagaacaaaaagaagataaaagccacttaatgacataccactctgcatcctgtgcaattgcaggtgaaggcgtggatattaatgatggaaaagcaattgcagaaggattattatcccaatttggctttacaaactttaaattcattcctgatgaaaagaaaagtaaatactacacccctgaaacacagactgaagtttacgcataccacccaaaattaaaagaatggctcgaagttgctacatttggagtatattcgccagttgcattaagcaaatacggaatagatgtacctgtaatgaatttgggtcttggtgttgaaagacttgcaatgatttctggaaatttcgcagatgttcgagaaatggtatatcctcagttttacgaacacaaacttaatgaccggaatgtcgcttcaatggtaaaactcgataaagttccagtaatggatgaaatttacgatttaacaaaagaattaattgagtcatgtgttaaaaacaaagatttaaaatccccttgtgaattagctattgaaaaaacgttttcatttggaaaaaccaagaaaaatgtaaaaataaacatttttgaaaaagaagaaggtaaaaatttactcggaccttcaattttaaacgaaatctacgtttacgatggaaatgtaattggaattcctgaaagctttgacggagtaaaagaagaatttaaagacttcttagaaaaaggaaaatcagaaggggtagcaacaggcattcgatatatcgatgcgctttgctttaaaattacttcaaaattagaagaagcatttgtgtcaaacactactgaattcaaagttaaagttccaattgtcagaagtttaagcgacattaacttaaaaatcgatgatatcgcattaaaacagatcatgagcaaaaataaagtaatcgacgttagaggcccagtctttttaaatgtcgaagtaaaaattgaataa |
| RS2 | atggcaatgtttaaaagagaagaaatcattgaaatggccaataaggactttgaaaaagcatggatcgaaactaaagaccttataaaagctaaaaagataaacgaaagttacccaagaataaaaccagtttttggaaaaacacaccctgtaaatgacactattgaaaatttaagacaggcatatcttagaatgggttttgaagaatatataaacccagtaattgtcgatgaaagagatatttataaacaattcggcccagaagctatggcagttttggatagatgcttttatttagcgggacttccaagacctgacgttggtttgagcgatgaaaaaatttcacagattgaaaaacttggaattaaagtttctgagcacaaagaaagtttacaaaaaatacttcacggatacaaaaaaggaactcttgatggtgacgatttagttttagaaatttcaaatgcacttgaaatttcaagcgagatgggtttaaaaattttagaagatgttttcccagaatttaaggatttaaccgcagtttcttcaaaattaactttaagaagccacatgacttcaggatggttccttactgtttcagacctcatgaacaaaaaacccttgccatttaaactcttttcaatcgatagatgttttagaagagaacaaaaagaagataaaagccacttaatgacataccactctgcatcctgtgcaattgcaggtgaaggcgtggatattaatgatggaaaagcaattgcagaaggattattatcccaatttggctttacaaactttaaattcattcctgatgaaaagaaaagtaaatactacacccctgaaacacagactgaagtttacgcataccacccaaaattaaaagaatggctcgaagttgctacatttggagtatattcgccagttgcattaagcaaatacggaatagatgtacctgtaatgaatttgggtcttggtgttgaaagacttgcaatgatttctggaaatttcgcagatgttcgagaaatggtatatcctcagttttacgaacacgaacttaatgaccgggatgtcgcttcaatggtaaaactcgataaagttccagtaatggatgaaatttacgatttaacaaaagaattaattgagtcatgtgttaaaaacaaagatttaaaatccccttgtgaattagctattgaaaaaacgttttcatttggaaaaaccaagaaaaatgtaaaaataaactttttttttaaaatagaaggtaaaaatttactcggaccttcaattttaaacgaaatctacgtttacgatggaaatgtaattggaattcctgaaagctttgacggagtaaaagaagaatttaaagacttcttagaaaaaggaaaatcagaaggggtagcaacaggcattcgatatatcgatgcgctttgctttaaaattacttcaaaattagaagaagcatttgtgtcaaacactactgaattcaaagttatgtggcgacgtgtcagaagtttaagcgacattaacttaaaaatcgatgatatcgcaataaaacagatcatgagcaaaaataaagtaatcgacgttagaggcagcgtctttttaaatgtcgaagtaaaaattgaaCACCACCACCACCACCACTGA |
| RS3 | atggcaatgtttaaaagagaagaaatcattgaaatggccaataaggactttgaaaaagcatggatcgaaactaaagaccttataaaagctaaaaagataaacgaaagttacccaagaataaaaccagtttttggaaaaacacaccctgtaaatgacactattgaaaatttaagacaggcatatcttagaatgggttttgaagaatatataaacccagtaattgtcgatgaaagagatatttataaacaattcggcccagaagctatggcagttttggatagatgcttttatttagcgggacttccaagacctgacgttggtttgagcgatgaaaaaatttcacagattgaaaaacttggaattaaagtttctgagcacaaagaaagtttacaaaaaatacttcacggatacaaaaaaggaactcttgatggtgacgatttagttttagaaatttcaaatgcacttgaaatttcaagcgagatgggtttaaaaattttagaagatgttttcccagaatttaaggatttaaccgcagtttcttcaaaattaactttaagaagccacatgacttcaggatggttccttactgtttcagacctcatgaacaaaaaacccttgccatttaaactcttttcaatcgatagatgttttagaagagaacaaaaagaagataaaagccacttaatgacataccactctgcatcctgtgcaattgcaggtgaaggcgtggatattaatgatggaaaagcaattgcagaaggattattatcccaatttggctttacaaactttaaattcattcctgatgaaaagaaaagtaaatactacacccctgaaacacagactgaagtttacgcataccacccaaaattaaaagaatggctcgaagttgctacatttggagtatattcgccagttgcattaagcaaatacggaatagatgtacctgtaatgaatttgggtcttggtgttgaaagacttgcaatgatttctggaaatttcgcagatgttcgagaaatggtatatcctcagttttacgaacacgaacttaatgaccgggatgtcgcttcaatggtaaaactcgataaagttccagtaatggatgaaatttacgatttaacaaaagaattaattgagtcatgtgttaaaaacaaagatttaaaatccccttgtgaattagctattgaaaaaacgttttcatttggaaaaaccaagaaaaatgtaaaaataaactttttttttaaaatacgtggtaaaaatttactcggaccttcaattttaaacgaaatctacgtttacgatggaaatgtaattggaattcctgaaagctttgacggagtaaaagaagaatttaaagacttcttagaaaaaggaaaatcagaaggggtagcaacaggcattcgatatatcgatgcgctttgctttaaaattacttcaaaattagaagaagcatttgtgtcaaacactactgaattcaaagttatgtggcgacgtgtcagaagtttaagcgacattaacttaaaaatcgatgatatcgcaataaaacagatcatgagcaaaaataaagtaatcgacgttagaggcagcgtctttttaaatgtcgaagtaaaaattgaaCACCACCACCACCACCACTGA |
| RS4 | atggcaatgtttaaaagagaagaaatcattgaaatggccaataaggactttgaaaaagcatggatcgaaactaaagaccttataaaagctaaaaagataaacgaaagttacccaagaataaaaccagtttttggaaaaacacaccctgtaaatgacactattgaaaatttaagacaggcatatcttagaatgggttttgaagaatatataaacccagtaattgtcgatgaaagagatatttataaacaattcggcccagaagctatggcagttttggatagatgcttttatttagcgggacttccaagacctgacgttggtttgagcgatgaaaaaatttcacagattgaaaaacttggaattaaagtttctgagcacaaagaaagtttacaaaaaatacttcacggatacaaaaaaggaactcttgatggtgacgatttagttttagaaatttcaaatgcacttgaaatttcaagcgagatgggtttaaaaattttagaagatgttttcccagaatttaaggatttaaccgcagtttcttcaaaattaactttaagaagccacatgacttcaggatggttccttactgtttcagacctcatgaacaaaaaacccttgccatttaaactcttttcaatcgatagatgttttagaagagaacaaaaagaagataaaagccacttaatgacataccactctgcatcctgtgcaattgcaggtgaaggcgtggatattaatgatggaaaagcaattgcagaaggattattatcccaatttggctttacaaactttaaattcattcctgatgaaaagaaaagtaaatactacacccctgaaacacagactgaagtttacgcataccacccaaaattaaaagaatggctcgaagttgctacatttggagtatattcgccagttgcattaagcaaatacggaatagatgtacctgtaatgaatttgggtcttggtgttgaaagacttgcaatgatttctggaaatttcgcagatgttcgagaaatggtatatcctcagttttacgaacacgaacttaatgaccgggatgtcgcttcaatggtaaaactcgataaagttccagtaatggatgaaatttacgatttaacaaaagaattaattgagtcatgtgttaaaaacaaagatttaaaatccccttgtgaattagctattgaaaaaacgttttcatttggaaaaaccaagaaaaatgtaaaaataaacttttttTTTaaaataTGGggtaaaaatttactcggaccttcaattttaaacgaaatctacgtttacgatggaaatgtaattggaattcctgaaagctttgacggagtaaaagaagaatttaaagacttcttagaaaaaggaaaatcagaaggggtagcaacaggcattcgatatatcgatgcgctttgctttaaaattacttcaaaattagaagaagcatttgtgtcaaacactactgaattcaaagttGCAGCAcgacgtgtcagaagtttaagcgacattaacttaaaaatcgatgatatcgcaataaaacagatcatgagcaaaaataaagtaatcgacgttagaggcGAAgtctttttaaatgtcgaagtaaaaattgaacaccaccaccaccaccactga |
| RS5 | atggcaatgtttaaaagagaagaaatcattgaaatggccaataaggactttgaaaaagcatggatcgaaactaaagaccttataaaagctaaaaagataaacgaaagttacccaagaataaaaccagtttttggaaaaacacaccctgtaaatgacactattgaaaatttaagacaggcatatcttagaatgggttttgaagaatatataaacccagtaattgtcgatgaaagagatatttataaacaattcggcccagaagctatggcagttttggatagatgcttttatttagcgggacttccaagacctgacgttggtttgagcgatgaaaaaatttcacagattgaaaaacttggaattaaagtttctgagcacaaagaaagtttacaaaaaatacttcacggatacaaaaaaggaactcttgatggtgacgatttagttttagaaatttcaaatgcacttgaaatttcaagcgagatgggtttaaaaattttagaagatgttttcccagaatttaaggatttaaccgcagtttcttcaaaattaactttaagaagccacatgacttcaggatggttccttactgtttcagacctcatgaacaaaaaacccttgccatttaaactcttttcaatcgatagatgttttagaagagaacaaaaagaagataaaagccacttaatgacataccactctgcatcctgtgcaattgcaggtgaaggcgtggatattaatgatggaaaagcaattgcagaaggattattatcccaatttggctttacaaactttaaattcattcctgatgaaaagaaaagtaaatactacacccctgaaacacagactgaagtttacgcataccacccaaaattaaaagaatggctcgaagttgctacatttggagtatattcgccagttgcattaagcaaatacggaatagatgtacctgtaatgaatttgggtcttggtgttgaaagacttgcaatgatttctggaaatttcgcagatgttcgagaaatggtatatcctcagttttacgaacacgaacttaatgaccgggatgtcgcttcaatggtaaaactcgataaagttccagtaatggatgaaatttacgatttaacaaaagaattaattgagtcatgtgttaaaaacaaagatttaaaatccccttgtgaattagctattgaaaaaacgttttcatttggaaaaaccaagaaaaatgtaaaaataaacTTTtttCCGaaaataAAAggtaaaaatttactcggaccttcaattttaaacgaaatctacgtttacgatggaaatgtaattggaattcctgaaagctttgacggagtaaaagaagaatttaaagacttcttagaaaaaggaaaatcagaaggggtagcaacaggcattcgatatatcgatgcgctttgctttaaaattacttcaaaattagaagaagcatttgtgtcaaacactactgaattcaaagttCCGTGGcgacgtgtcagaagtttaagcgacattaacttaaaaatcgatgatatcgcaataaaacagatcatgagcaaaaataaagtaatcgacgttagaggcCATgtctttttaaatgtcgaagtaaaaattgaacaccaccaccaccaccactga |
| RS6 | atggacgaatttgaaatgataaagagaaacacatctgaaattatcagcgaggaagagttaagagaggttttaaaaaaagatgaaaaatctgctggtataggttttgaaccaagtggtaaaatacatttagggcattatctccaaataaaaaagatgattgatttacaaaatgctggatttgatataattatattgttggctgatttacacgcctatttaaaccagaaaggagagttggatgagattagaaaaataggagattataacaaaaaagtttttgaagcaatggggttaaaggcaaaatatctttatggaagtcctttccagcttgataaggattatacactgaatgtctatagattggctttaaaaactaccttaaaaagagcaagaaggagtatggaacttatagcaagagaggatgaaaatccaaaggttgctgaagttatctatccaataatgcaggttaatacgagtcattatctgggcgttgatgttgcagttggagggatggagcagagaaaaatacacatgttagcaagggagcttttaccaaaaaaggttgtttgtattcacaaccctgtcttaacgggtttggatggagaaggaaagatgagttcttcaaaagggaattttatagctgttgatgactctccagaagagattagggctaagataaagaaagcatactgcccagctggagttgttgaaggaaatccaataatggagatagctaaatacttccttgaatatcctttaaccataaaaaggccagaaaaatttggtggagatttgacagttaatagctatgaggagttagagagtttatttaaaaataaggaattgcatccaatggatttaaaaaatgctgtagctgaagaacttataaagattttagagccaattagaaagagattataa |
| RS7 | atggacgagttcgaaatgattaaacgcaacaccagcgaaattatctctgaagaagagctgcgcgaggtgctgaagaaagacgagaagagcgcgctgattggctttgagccgtccggtaaaattcacctgggtcactacctgcaaatcaagaagatgattgatctgcaaaacgctggttttgacatcattatcctgctggcggacctgcacgcctacctgaatcaaaagggcgagctggatgagattcgcaagatcggcgactacaataagaaagtcttcgaagccatgggtttgaaggctaaatacgtctacggtagcGAAtttcagctggataaggattacacgttgaatgtgtaccgtctggcgctgaaaaccacgctgaaacgcgcccgtcgttccatggagctgattgcgcgcgaggatgagaatccaaaagttgctgaggttatttaccctattatgcaagttaatGGTTGTcactacAGGggtgttgatgttgccgtcggtggtatggagcaacgcaaaattcacatgctggcacgtgaactgctgccgaaaaaggttgtctgtattcataatccggtcctgaccggcctggatggcgagggtaaaatgagcagcagcaagggtaactttattgcagttgacgatagcccggaagaaatccgtgcgaagatcaagaaagcgtactgcccggcaggcgtggttgagggtaacccgatcatggaaatcgccaagtattttctggaatacccactgacgattaagcgcccggagaaatttggcggcgacctgaccgtcaacagctacgaggagctggaaagcttgtttaagaacaaagaactgcatccgatgcgcctgaaaaacgccgtggcggaagagctgattaagattctggaaccaattcgcaaacgtctgtaa |
| RS8 | atggacgagttcgaaatgattaaacgcaacaccagcgaaattatctctgaagaagagctgcgcgaggtgctgaagaaagacgagaagagcgcgactattggctttgagccgtccggtaaaattcacctgggtcactacctgcaaatcaagaagatgattgatctgcaaaacgctggttttgacatcattatcctgctggcggacctgcacgcctacctgaatcaaaagggcgagctggatgagattcgcaagatcggcgactacaataagaaagtcttcgaagccatgggtttgaaggctaaatacgtctacggtagcaattttcagctggataaggattacacgttgaatgtgtaccgtctggcgctgaaaaccacgctgaaacgcgcccgtcgttccatggagctgattgcgcgcgaggatgagaatccaaaagttgctgaggttatttaccctattatgcaagttaatccgttgcactaccagggtgttgatgttgccgtcggtggtatggagcaacgcaaaattcacatgctggcacgtgaactgctgccgaaaaaggttgtctgtattcataatccggtcctgaccggcctggatggcgagggtaaaatgagcagcagcaagggtaactttattgcagttgacgatagcccggaagaaatccgtgcgaagatcaagaaagcgtactgcccggcaggcgtggttgagggtaacccgatcatggaaatcgccaagtattttctggaatacccactgacgattaagcgcccggagaaatttggcggcgacctgaccgtcaacagctacgaggagctggaaagcttgtttaagaacaaagaactgcatccgatgcgcctgaaaaacgccgtggcggaagagctgattaagattctggaaccaattcgcaaacgtctgtaa |
| RS9 | atggacgagttcgaaatgattaaacgcaacaccagcgaaattatctctgaagaagagctgcgcgaggtgctgaagaaagacgagaagagcgcgTATattggctttgagccgtccggtaaaattcacctgggtcactacctgcaaatcaagaagatgattgatctgcaaaacgctggttttgacatcattatcctgctggcggacctgcacgcctacctgaatcaaaagggcgagctggatgagattcgcaagatcggcgactacaataagaaagtcttcgaagccatgggtttgaaggctaaatacgtctacggtagcGAAtttcagctggataaggattacacgttgaatgtgtaccgtctggcgctgaaaaccacgctgaaacgcgcccgtcgttccatggagctgattgcgcgcgaggatgagaatccaaaagttgctgaggttatttaccctattatgcaagttaatGATATTcactacCTGggtgttgatgttgccgtcggtggtatggagcaacgcaaaattcacatgctggcacgtgaactgctgccgaaaaaggttgtctgtattcataatccggtcctgaccggcctggatggcgagggtaaaatgagcagcagcaagggtaactttattgcagttgacgatagcccggaagaaatccgtgcgaagatcaagaaagcgtactgcccggcaggcgtggttgagggtaacccgatcatggaaatcgccaagtattttctggaatacccactgacgattaagcgcccggagaaatttggcggcgacctgaccgtcaacagctacgaggagctggaaagcttgtttaagaacaaagaactgcatccgatgGATctgaaaaacgccgtggcggaagagctgattaagattctggaaccaattcgcaaacgtctgtaa |
| RS10 | cgttgagtttctccagaagcgttaatgtctggcttctgataaagcgggccatgttaagggcggttttttcctgtttggtcactgatgcctccgtgtaagggggatttctgttcatgggggtaatgataccgatgaaacgagagaggatgctcacgatacgggttactgatgatgaacatgcccggttactggaacgttgtgagggtaaacaactggcggtatggatgcggcgggaccagagaaaaatcactcagggtcaatgccagcgcttcgttaatacagatgtaggtgttccacagggtagccagcagcatcctgcgatgcagatccggaacataatggtgcagggcgctgacttccgcgtttccagactttacgaaacacggaaaccgaagaccattcatgttgttgctcaggtcgcagacgttttgcagcagcagtcgcttcacgttcgctcgcgtatcggtgattcattctgctaaccagtaaggcaaccccgccagcctagccgggtcctcaacgacaggagcacgatcatgcgcacccgtggccaggacccaacgctgcccgagatctcgatcccgcgaaattaatacgactcactatagggagaccacaacggtttccctctagaaataattttgtttaactttaagaaggagatatacatatggacgagttcgaaatgattaaacgcaacaccagcgaaattatctctgaagaagagctgcgcgaggtgctgaagaaagacgagaagagcgcgtatattggctttgagccgtccggtaaaattcacctgggtcactacctgcaaatcaagaagatgattgatctgcaaaacgctggttttgacatcattatcctgctggcggacctgcacgcctacctgaatcaaaagggcgagctggatgagattcgcaagatcggcgactacaataagaaagtcttcgaagccatgggtttgaaggctaaatacgtctacggtagcgaatttcagctggataaggattacacgttgaatgtgtaccgtctggcgctgaaaaccacgctgaaacgcgcccgtcgttccatggagctgattgcgcgcgaggatgagaatccaaaagttgctgaggttatttaccctattatgcaagttaatgatattcactacctgggtgttgatgttgccgtcggtggtatggagcaacgcaaaattcacatgctggcacgtgaactgctgccgaaaaaggttgtctgtattcataatccggtcctgaccggcctggatggcgagggtaaaatgagcagcagcaagggtaactttattgcagttgacgatagcccggaagaaatccgtgcgaagatcaagaaagcgtactgcccggcaggcgtggttgagggtaacccgatcatggaaatcgccaagtattttctggaatacccactgacgattaagcgcccggagaaatttggcggcgacctgaccgtcaacagctacgaggagctggaaagcttgtttaagaacaaagaactgcatccgatggatctgaaaaacgccgtggcggaagagctgattaagattctggaaccaattcgcaaacgt |
| RS11 | tacatatggataaaaaaccactaaacactctgatctctgctactggtctgtggatgagtcgtaccggaaccattcataaaatcaaacaccacgaggttagccgttcgaaaatctatattgagatggcgtgtggcgatcatctggttgtgaacaatagccgctcttctcgtacagcacgtgcactgcgtcaccacaaatatcgtaaaacctgtaaacgttgccgtgtgtccgatgaggatctgaacaaattcctgacaaaagccaatgaggaccaaacaagcgtgaaagtgaaagtcgttagcgctcctacccgtactaaaaaagcaatgccgaaatccgttgctcgtgcccctaaaccactggaaaacactgaagcagcacaggcacagccgtctggaagcaaattctctccggccattcctgtttctacccaggagtccgtttctgttccagcaagtgtgagcaccagcattagcagtattagcaccggtgccaccgctagcgccctggttaaaggcaataccaatccgattacaagcatgtctgccccggttcaagcatcagctccagcactgacaaaatcccaaaccgatcgtctggaggttctgctgaatccgaaagacgaaatcagcctgaattccggcaaaccgtttcgtgaactggagagcgaactgctgtcacgtcgtaaaaaagacctgcaacaaatctatgccgaagaacgtgagaactatctggggaaactggaacgtgaaatcacccgctttttcgtggatcgtggctttctggagatcaaatccccgattctgattcctctggagtatatcgagcgtatgggcatcgacaatgataccgaactgagcaaacaaattttccgtgtggataaaaacttctgtctgcgccctatgctgtctccaaatctgtataactatatgcgcaaactggaccgtgccctgcctgatcctatcaaattgttcgagatcggcccgtgttatcgtaaagagtccgacggtaaagaacatctggaggagtttaccatgctggcttttggtcaaatgggttcaggttgtactcgtgagaacctggaaagcatcatcaccgattttctgaaccacctgggcattgacttcaaaattgtgggcgacagctgtatggtgtatggcgacaccctggatgtcatgcacggcgacctggaactgtctagtgccgttgttggaccaattccgctggaccgtgagtggggtatcgacaaaccgacgatcggagcaggattcggtctggaacgcctgctgaaagtgaaacacgacttcaaaaacatcaaacgtgccgcccgttctgaatcgtattataacgggatttctaccaacctg |

**Table S5** Incorporation analysis of unnatural amino acid O-pSer by mass spectrometry

| Incorporated AA | | Peptide fragments | PSMs^1^ | Ratio^2^ |
| --- | --- | --- | --- | --- |
| Unnatural | O-pSer | KGEELFTGVVPILVELDGDV**(O-pSer)**GHK | 7 | 2.72% |
| Natural | Q | KGEELFTGVVPILVELDGDV**Q**GHK | 126 | 49.03% |
|  | K | KGEELFTGVVPILVELDGDV**K**GHK | 61 | 23.74% |
|  | Y | KGEELFTGVVPILVELDGDV**Y**GHK | 36 | 14.01% |
|  | C | KGEELFTGVVPILVELDGDV**C**GHK | 8 | 3.11% |
|  | I | KGEELFTGVVPILVELDGDV**I**GHK | 9 | 3.50% |
|  | N | KGEELFTGVVPILVELDGDV**N**GHK | 3 | 1.17% |
|  | F | KGEELFTGVVPILVELDGDV**F**GHK | 2 | 0.78% |
|  | M | KGEELFTGVVPILVELDGDV**M**GHK | 2 | 0.78% |
|  | V | KGEELFTGVVPILVELDGDV**V**GHK | 1 | 0.39% |
|  | P | KGEELFTGVVPILVELDGDV**P**GHK | 1 | 0.39% |
|  | T | KGEELFTGVVPILVELDGDV**T**GHK | 1 | 0.39% |

Note:

^1^PSMs means the number of peptide spectrum matches. The number of PSMs is the total number of identified peptide spectra matched for the protein.

^2^The incorporation ratio of certain amino acid = The PSMs value of incorporated certain amino acid/The total PSMs value of all incorporated amino acids*100%.

**Table S6** Incorporation analysis of unnatural amino acid C-pSer by mass spectrometry

| Incorporated AA | | Peptide fragments | PSMs^1^ | Ratio^2^ |
| --- | --- | --- | --- | --- |
| Unnatural | C-pSer | KGEELFTGVVPILVELDGDV**(C-pSer)**GHK | 1 | 0.80% |
| Natural | Q | KGEELFTGVVPILVELDGDV**Q**GHK | 63 | 50.40% |
|  | K | KGEELFTGVVPILVELDGDV**K**GHK | 32 | 25.60% |
|  | Y | KGEELFTGVVPILVELDGDV**Y**GHK | 18 | 14.40% |
|  | C | KGEELFTGVVPILVELDGDV**C**GHK | 3 | 2.40% |
|  | I | KGEELFTGVVPILVELDGDV**I**GHK | 4 | 3.20% |
|  | W | KGEELFTGVVPILVELDGDV**W**GHK | 2 | 1.60% |
|  | N | KGEELFTGVVPILVELDGDV**N**GHK | 2 | 1.60% |

Note:

^1^PSMs means the number of peptide spectrum matches. The number of PSMs is the total number of identified peptide spectra matched for the protein.

^2^The incorporation ratio of certain amino acid = The PSMs value of incorporated certain amino acid/The total PSMs value of all incorporated amino acids*100%.

# Supplementary Figures


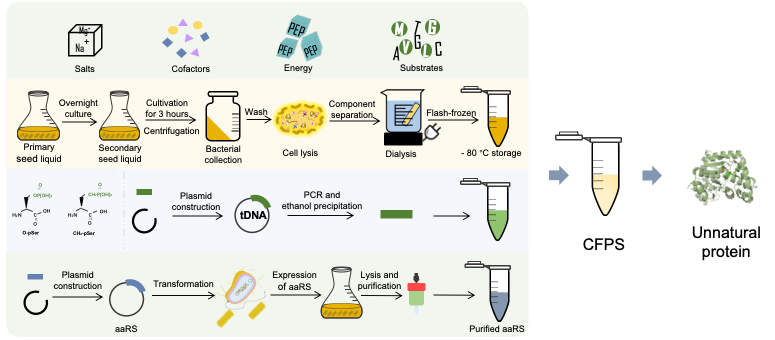


**Figure S1.** The flow diagram of cell-free protein synthesis (CFPS) reaction operation

**Figure S2.** The screening results of magnesium ion concentrations in the CFPS system. The charges produced by the nucleic acid phosphate groups and other anionic species in the system can be balanced using Mg^2+^. Additionally, it can influence how proteins and nucleic acids interact during biological processes like protein synthesis and can act as an enzyme activator for particular enzymes like RNA polymerase. It was found that the concentration of Mg^2+^ had a combined influence on the expression of sfGFP in CFPS systems.

**Figure S3.** The time profile of the CFPS reaction without OTS

**Figure S4.** The time profile of the CFPS reaction with OTS


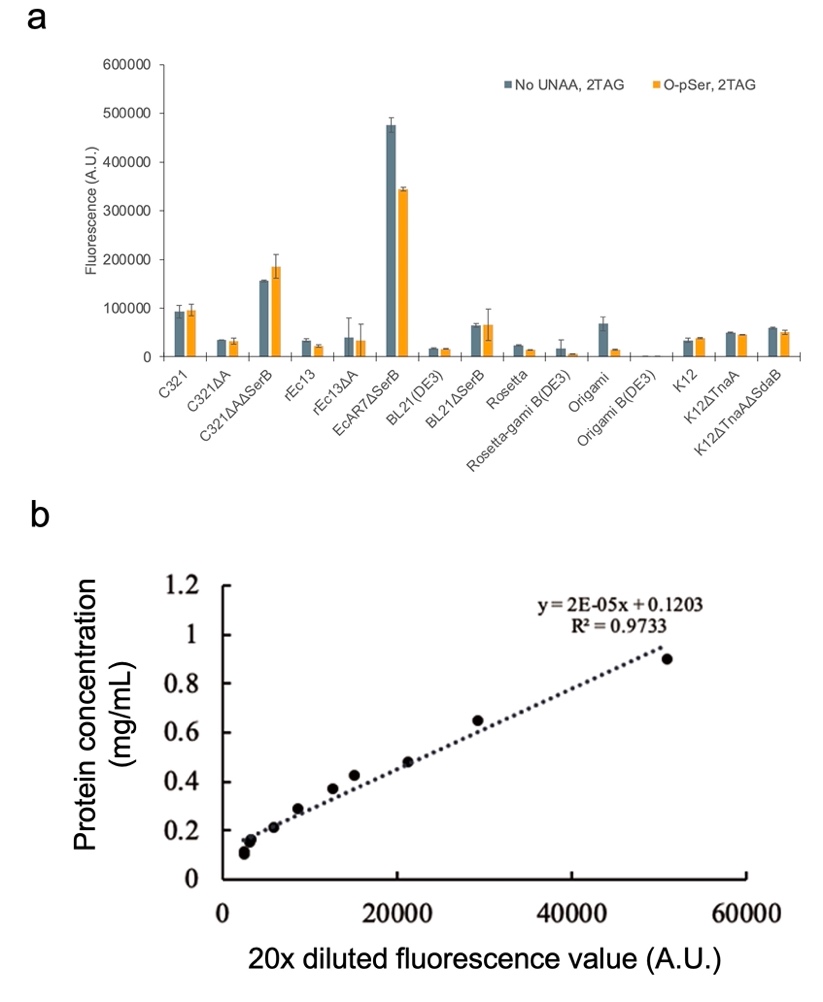


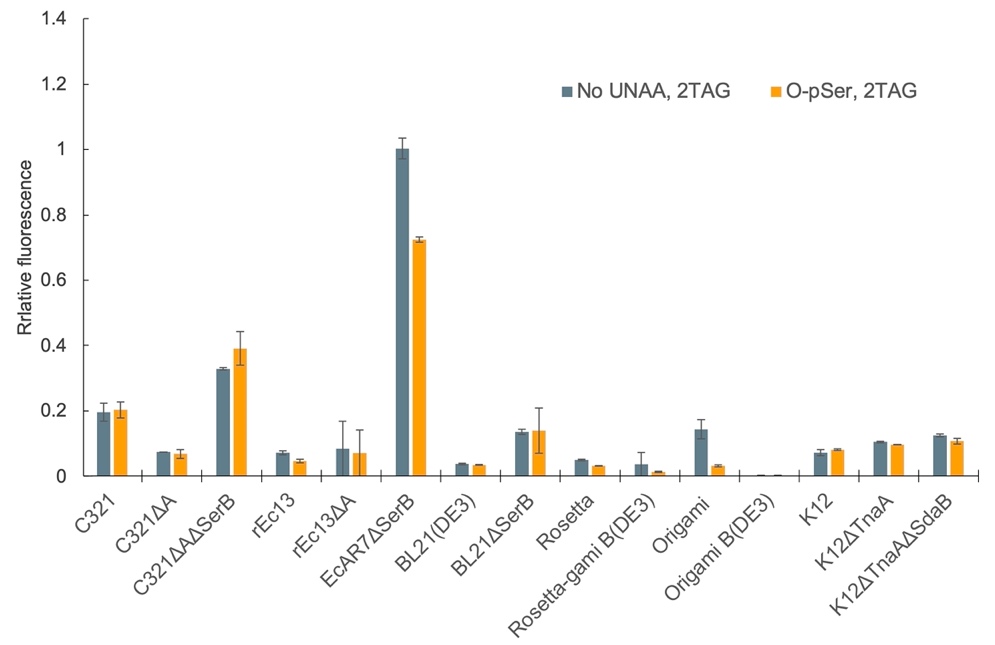


**Figure S5.** Chassis cell screening for O-pSer incorporation in 2TAG-sfGFP. When the relative fluorescence was 1, the corresponding protein expression level was 0.60 mg/mL.

(a) Screening results. (b) The fluorescent value could represent the protein expression level. The sample was diluted 20 times. The fluorescent value of sfGFP increased linearly with the concentration of protein, which indicated that the protein expression level could be characterized by the fluorescence


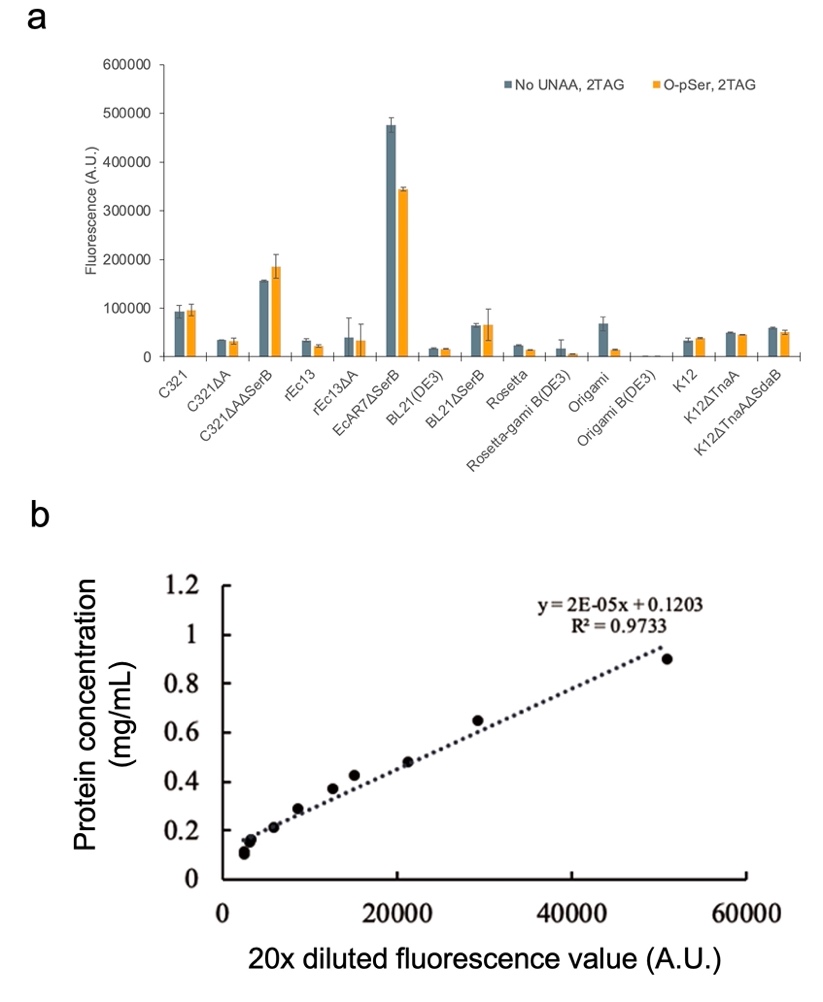


**Figure S6.** The fluorescent value could represent the protein expression level. The sample was diluted 20 times. The fluorescent value of sfGFP increased linearly with the concentration of protein, which indicated that the protein expression level could be characterized by the fluorescence

**Figure S7.** Chassis cell screening and comparison for O-pSer and C-pSer incorporation in 2TAG-sfGFP


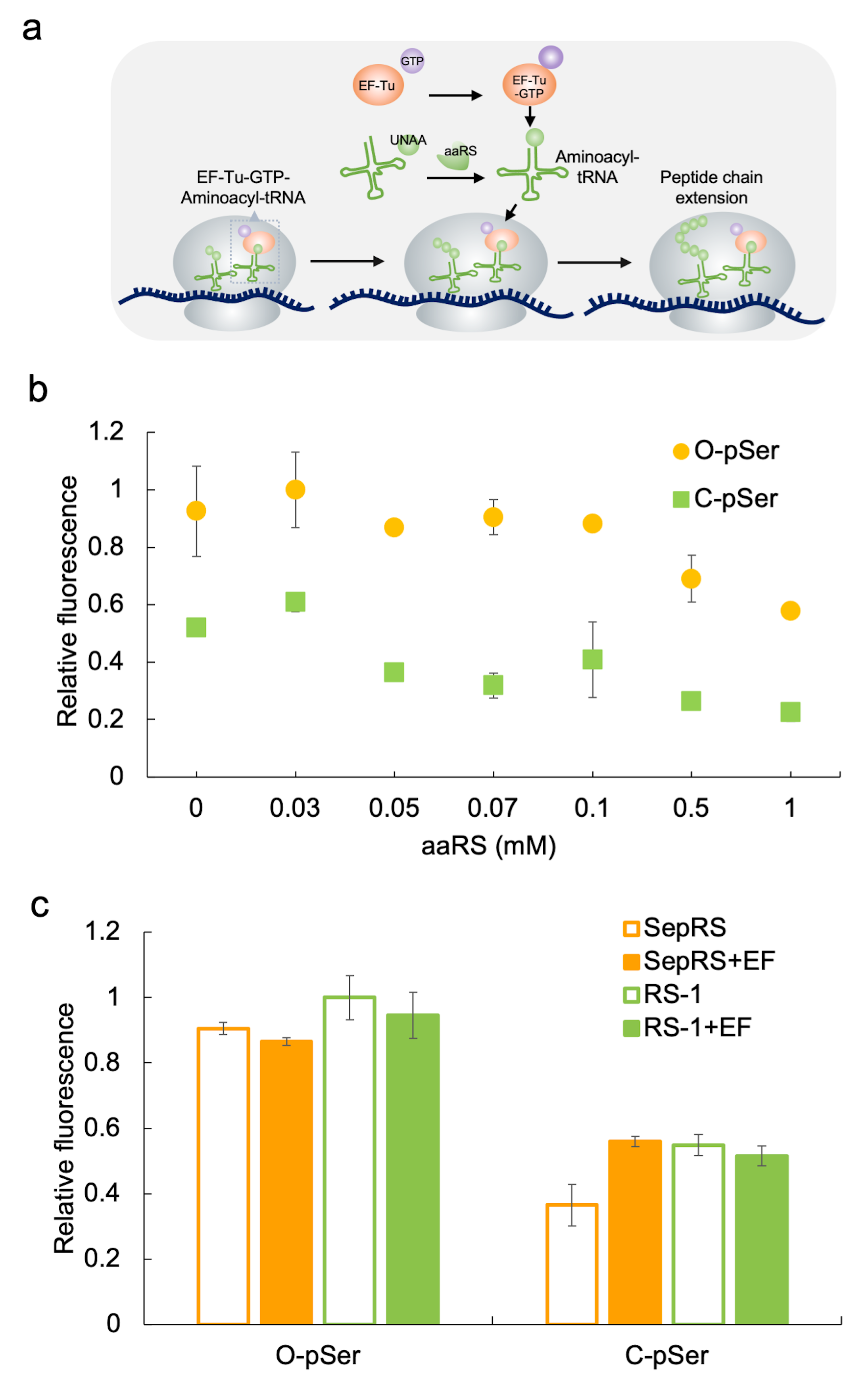


**Figure S8. Exogenous OTS addition methods for precise cell-free regulation.** (a) Schematic of the process of protein expression with the proper running of essential protein translation machinery. (b) Time-course and endpoint analysis of the aaRS concentration added into CFPS for sfGFP synthesis in the presence of the Sep-OTS. A TAG codon at position 23 directed O-pSer and C-pSer incorporation into sfGFP. (c) The effect of EF-Tu addition on sfGFP expression level. A TAG codon at position 23 directed O-pSer and C-pSer incorporation into sfGFP, and a control with no EF-Tu addition was used. Expression level was determined by fluorescence intensity of generated sfGFP protein. Error bars report SD from three biological replicates.


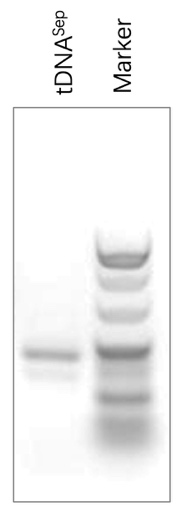


**Figure S9.** DNA gel analysis of purified tDNA

**Figure S10.** Purification results of proteins expressed in cells

**Figure S11.** Screening results of aaRS addition concentration in the CFPS systems

**Figure S12.** Screening results of tDNA addition concentration in the CFPS systems

**Figure S13.** Screening results of EF-TU addition in the CFPS systems with OTSs

**Figure S14.** Screening and comparison of EF-TU addition in the CFPS systems with OTSs

**Figure S15.** The structure and sequence of aaRS variants. (a) Structures of aaRS variants are shown in green cartoon and the side chains of mutation residues on aaRS binding pocket are indicated. Figures were created using PyMOL. (b) Sequence comparison of aaRS variants was shown.

**Figure S16.** Sequence library of selected tRNAs variants. Bases that remain unchanged in the selected clones are shown in black, while those that were mutated are shown in red.

**Figure S17.** The orthogonality analysis for reconfigured OTS components (aaRS and tRNA for O-pSer). The heatmap depicted the fluorescence value of the expressed sfGFP (UNAA-added CFPS reaction).

**Figure S18.** The orthogonality analysis for reconfigured OTS components (aaRS and tRNA for C-pSer). The heatmap depicted the fluorescence value of the expressed sfGFP (UNAA-added CFPS reaction).

**Figure S19.** The heatmap of the fluorescence value of the expressed sfGFP in the CFPS systems without UNAA addition.

**Figure S20.** SDS-PAGE results of purified proteins for mass spectrometry analysis

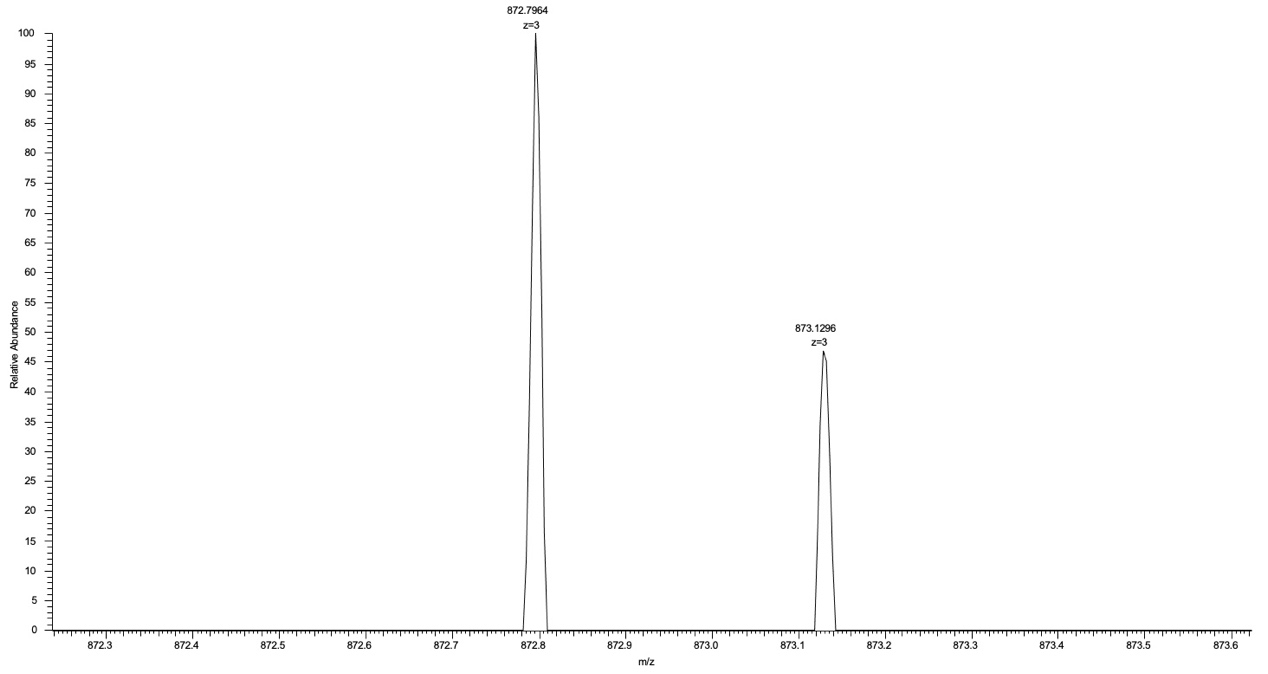


**Figure S21.** Mass spectrometry analysis results of O-pSer incorporation


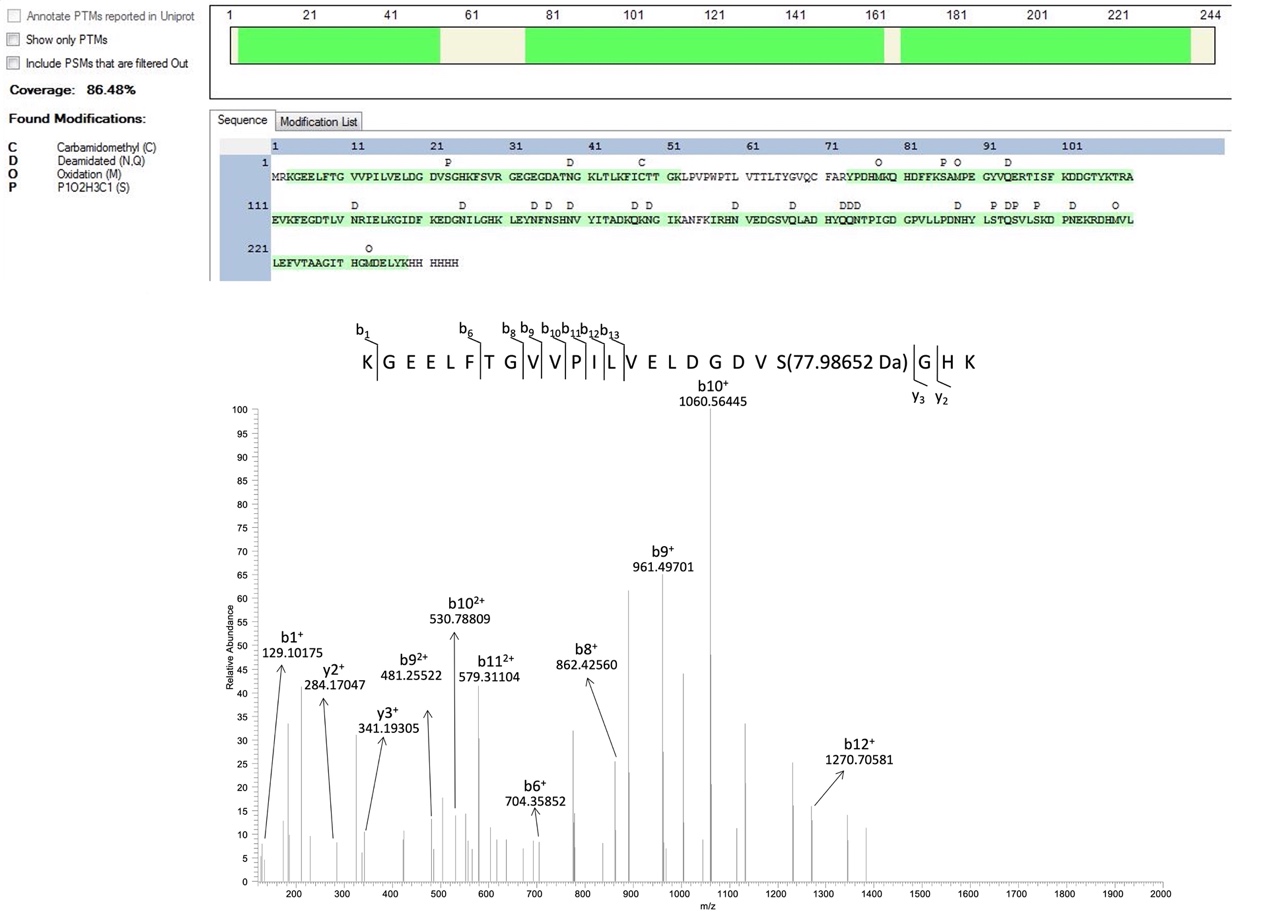


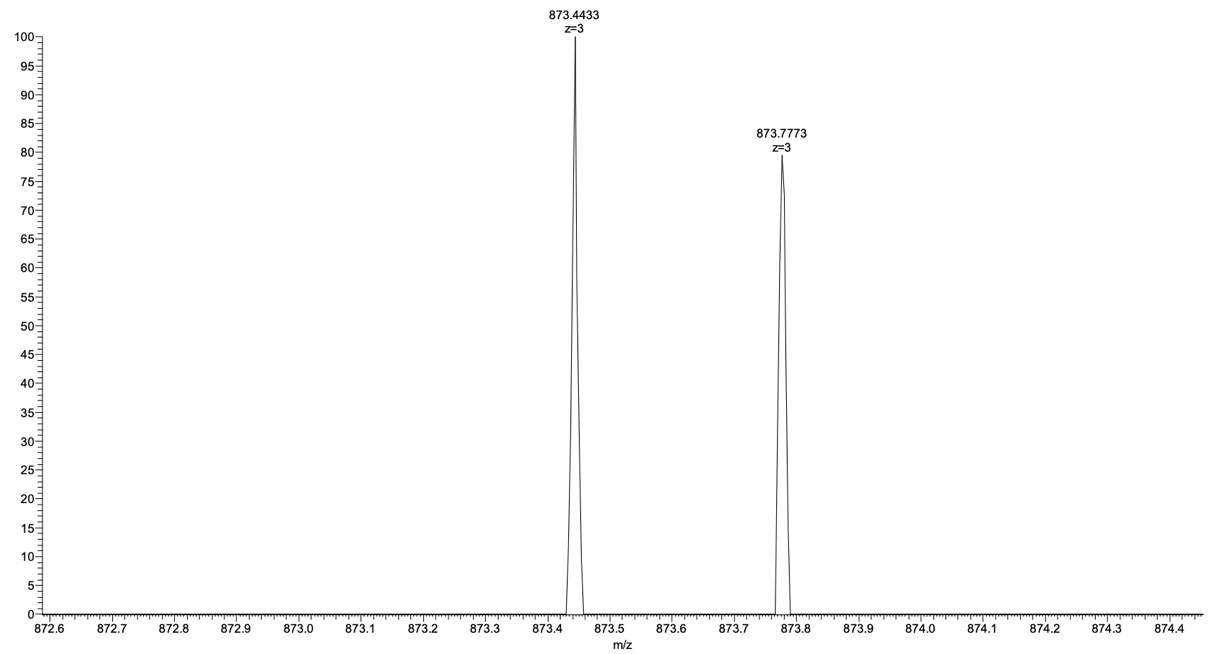


**Figure S22.** Mass spectrometry analysis results of C-pSer incorporation


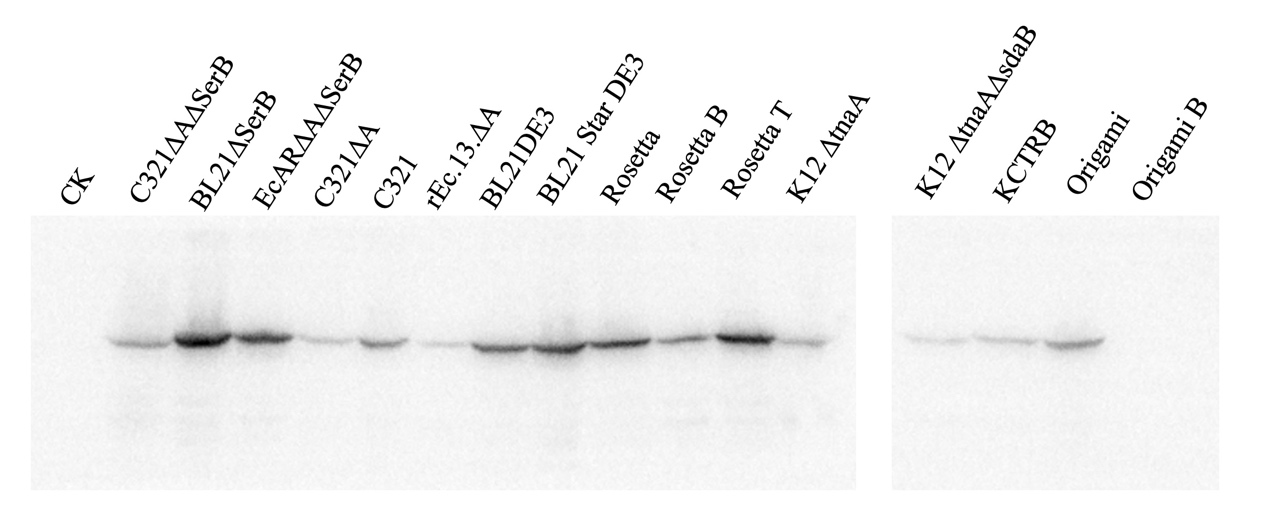


**Figure S23.** Western blot analysis of the MEK1 expression in the CFPS systems based on different chassis cells


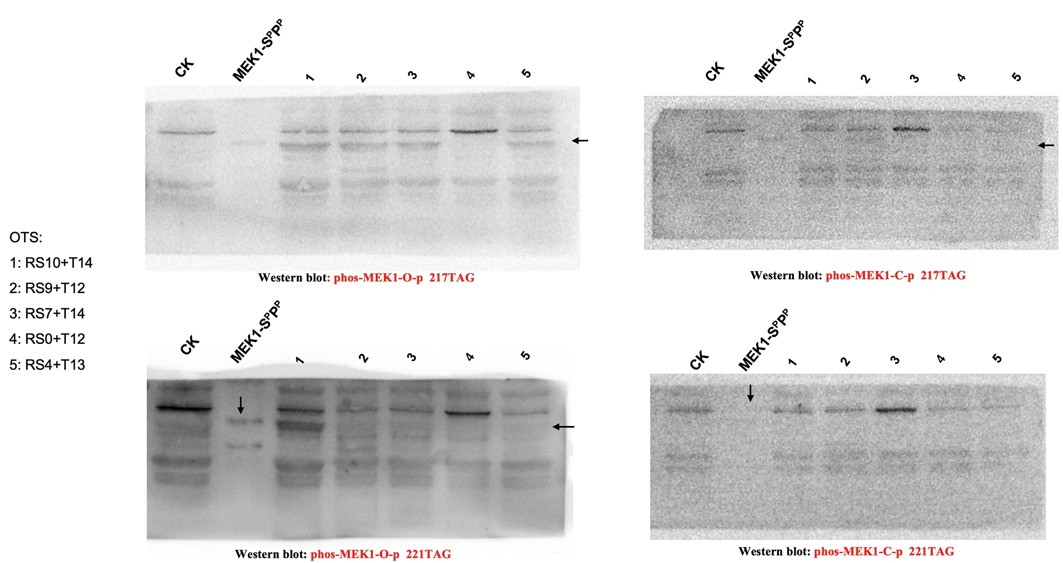


**Figure S24.** Western blot analysis of the phosphorylated MEK1 after site-specific UNAA incorporation
